# OSBPL10 Drives Lipophagy-Mediated Lipid Mobilization to Promote Pancreatic Ductal Adenocarcinoma Progression

**DOI:** 10.7150/ijbs.125552

**Published:** 2026-03-25

**Authors:** Zonghao Duan, Xueshiyu Ma, Feng Yu, Junfeng Zhang, Rong Hua, Wei Liu, Dejun Liu, Jianyu Yang, Xueliang Fu, Minwei Yang, Hongfei Yao, Shuheng Jiang, Lipeng Hu, Musitaba Mutailifu, Xiaomei Yang, Yongwei Sun, Linli Yao, Yanmiao Huo

**Affiliations:** 1 Department of Biliary-Pancreatic Surgery, Ren Ji Hospital, Shanghai Jiao Tong University School of Medicine, Shanghai, 200127, China.; 2 Department of General Surgery, Pancreatic Disease Center, Research Institute of Pancreatic Diseases, Ruijin Hospital, Shanghai Jiao Tong University School of Medicine, Shanghai, 200025, China.; 3 Shanghai Key Laboratory of Pancreatic Neoplasms Translational Research, Research Institute of Pancreatic Disease, Shanghai Jiao Tong University School of Medicine, Shanghai, China.; 4 State Key Laboratory of Systems Medicine for Cancer, Shanghai Cancer Institute, Ren Ji Hospital, School of Medicine, Shanghai Jiao Tong University, Shanghai, 200240, China.; 5 Department of Hepato-Biliary-Pancreatic Surgery, Huadong Hospital Affiliated to Fudan University, Shanghai, 200040, China.; 6 Shanghai Key Laboratory of Cancer Systems Regulation and Clinical Translation, Jiading District Central Hospital Affiliated Shanghai University of Medicine & Health Science, Shanghai, 201800, China.

**Keywords:** PDAC, lipophagy, rapid lysosomal repair, lipid metabolism, autophagy

## Abstract

Pancreatic ductal adenocarcinoma (PDAC) is an aggressive malignancy with a poor prognosis, in which the role of lipophagy, a selective autophagic process degrading lipid droplets (LDs), remains poorly characterized. This study investigated lipophagy and its key regulator, *OSBPL10*, in PDAC progression. Through immunofluorescence analysis of patient samples, transgenic mouse tissues, and cell lines, we find that lipophagy is elevated in PDAC and correlates with poor prognosis. Single-cell transcriptomic analysis identified *OSBPL10* as a critical lipophagy regulator and an independent clinicopathological indicator. Functional assays, including orthotopic and subcutaneous xenografts, demonstrated that *OSBPL10* promotes tumor growth. Mechanistically, OSBPL10 functionally cooperates with VAPA/VAPB to facilitate rapid lysosomal repair via ATG2A, thereby promoting lipophagy and lipid mobilization. Inhibition of lysosomal function abrogated the pro-lipophagic and pro-tumorigenic effects of OSBPL10. Collectively, our findings demonstrate that upregulated *OSBPL10* drives PDAC progression by enhancing lipophagy through ATG2A-mediated rapid lysosomal repair, highlighting OSBPL10 as a potential therapeutic target in PDAC.

## Introduction

Metabolic reprogramming is a hallmark of cancer, contributing significantly to tumorigenesis by providing essential nutrients for tumor cell proliferation 1, 2. Tumor cells often possess altered metabolic pathways, which supports key aspects of malignancy, including proliferation, migration, and invasion 3. LDs, cellular organelles that are central to lipid metabolism, are also involved in this metabolic shift 4. Traditionally, LDs serve as intracellular depots for triacylglycerols (TGs) and sterol esters in most mammalian cells. However, in tumor cells, LDs undergo mobilization and utilization in a more dynamic and unorthodox manner to support aggressive tumor growth. Lipids are released and utilized to fuel critical biological processes, such as providing energy for cancer cells, contributing to chemoresistance, and supporting invasive and migratory behavior 5, 6. Therefore, the mobilization and utilization of LDs are integral to the metabolic reprogramming that underpins cancer progression.

While lipolysis has long been recognized as a key process in lipid metabolism, in which free fatty acids are rapidly deployed for energy through β-oxidation, lipophagy is being gradually focused on 7. Lipophagy is a form of selective autophagy that degrades intracellular LDs via lysosomal action, which has drawn increasing attention for its role in modulating lipid storage 8. Recent studies have highlighted the complexity of lipophagy, with both chaperone-mediated lipophagy and macroautophagy observed in specific contexts. Unlike lipolysis, which typically breaks down LDs into free fatty acids, lipophagy appears to act as an upstream regulator, breaking down large LDs into smaller vesicles, thus facilitating more efficient lipid turnover and supporting rapid tumor cell metabolism 9, 10. This suggests that lipophagy plays an essential role in maintaining lipid homeostasis in cancer cells by regulating the availability of lipid-derived nutrients required for tumor progression.

The oxysterol-binding protein-like 8 (OSBPL8), also known as ORP8, has been implicated as a receptor for lipophagy. It belongs to the oxysterol-binding protein (OSBP) family, which plays a pivotal role in intracellular lipid trafficking and signal transduction between organelles 8. Another study by Chen *et al.* also unveiled the mechanism of lipophagy and fatty acid β-oxidation in acetaminophen-induced hepatotoxicity in mice 11. Moreover, a recent study by Kang *et al.* displayed methods to evaluate lipophagy and integrated the autophagy-dependent and -independent lipophagy in yeast, providing further evidence for this intriguing phenomenon 12. However, with such attention to lipophagy, its occurrence and effect in cancers, especially in PDAC, have yet to be explored.

Pancreatic cancer, a deadly malignant tumor, is universally acknowledged as a “silent killer” to suffering patients due to its insidious onset to early-stage detection and high mortality rate 13, 14. As the incidence is gradually increasing by 1% per year, pancreatic cancer currently leads third place in the United States and the seventh in the world in terms of cancer death, drawing much more attention nowadays. PDAC accounts for about 90% of pancreatic cancer, and as the cancer type with the highest degree of malignancy, PDAC is also known for its poor sensitivity to multiple treatments 5, 15, 16. Hence, the intense need to discover the reason of the high recurrence and mortality rates of PDAC is a necessity, which requires a deeper understanding of its malignant features 17. Tumor cells need an enormous amount of metabolites to support the need for proliferation to meet the need for its fast progression, and it has already been reported that one of the key enzymes of fatty acid biosynthesis, FASN, is upregulated and plays a pivotal role in PDAC progression 18. Moreover, synthesis-related enzymes such as ACLY also proved to be significant in PDAC cells 19.

Oxysterol binding protein like 10 (OSBPL10), as well as OSBPL8, is also part of the oxysterol-binding protein family, a group of pivotal transporters of signals between organelles and molecules 8. The particular family also participates in the regulation of the actin cytoskeleton, cell polarity, and cell itself 20. In a recent study, Tan *et al.* revealed a basilic role of OSBPL10 in anchoring damaged lysosomes onto the endoplasmic reticulum and therefore facilitating their quick repair 21. Based on previous studies of reprogrammed lipid metabolism in PDAC, we speculated that there might be a potential mechanism for tumor cells to quickly mobilize intracellular lipids due to their urgent need for proliferation and migration and provide flexibility to tumor cells to adjust according to its nutrition level at their microenvironment no matter high or low 22, 23.

Here, we demonstrate that OSBPL10 regulates lipophagy in PDAC both *in vitro* and *in vivo*, and its expression is positively correlated with poor clinical outcomes in PDAC patients. Furthermore, we elucidate the mechanism by which OSBPL10 promotes PDAC progression by enhancing of rapid lysosomal repair and lipid mobilization, thereby supporting the tumor aggressive growth.

## Materials and Methods

### Cell culture and reagents

The human PDAC cell lines SW1990, PANC-1, Patu8988, Capan-1, and other cell lines were purchased from the Cell Bank of the Chinese Academy of Sciences (Shanghai, China), and the murine PDAC cell line KPC1199 was maintained at Shanghai Cancer Institute, Ren Ji Hospital, School of Medicine, Shanghai Jiao Tong University. All cells were cultured in Dulbecco's modified Eagle's medium (DMEM) or Roswell Park Memorial Institute 1640 medium with 10% FBS and 1% penicillin/streptomycin (P/S) at 37°C in a humidified incubator with 5% CO2, according to the suggestions of American Type Culture Collection (ATCC) protocols.

### PDAC transgenic model, orthotopic xenograft model and subcutaneous xenograft model

All procedures were conducted in compliance with the approved protocol (A2020108) from the Research Ethics Committee of Shanghai Jiao Tong University. The mouse studies were conducted in compliance with the National Institutes of Health Guide for the Care and Use of Laboratory Animals. Experimental groups were assigned using stratified randomization based on the body weights of the animals.

The PDAC transgenic mouse model was established by crossing Pdx1-Cre mice with lox-stop-lox-Kras^G12D/+^ and lox-stop-lox-Trp53^R172H/+^ mice (KPC) to better elucidate the process of PDAC carcinogenesis. Pancreatic intraepithelial neoplasia (PanIN) lesions were generated in a cohort of lox-stop-lox-Kras^G12D/+^ and Pdx1-Cre (KC) mice 24. Pancreatic tissues from KPC mice were collected once tumors became palpable. KC mice were sacrificed at 18 and 36 weeks of age for the collection of early and late PanIN lesions, respectively.

For the orthotopic xenograft model, 1.5 × 10^6^ *Osbpl10^KD^* KPC1199^luc^ cells in 20 μL were transplanted into the pancreas of mice. The corresponding negative control cells were also transplanted in parallel to observe tumor growth. All mice were injected with D-luciferin and imaged for bioluminescence imaging under 2.5% isoflurane inhalation anesthesia. Mice were sacrificed 4 weeks post-implantation, and the tumor tissues were collected, fixed in 4% Paraformaldehyde Fix Solution (4% PFS), weighed, and photographed.

For the subcutaneous xenograft model, 2.0 × 10^6^ *OSBPL10^OE^* Patu8988 cells and the same number of cells of negative control were resuspended in 50μL PBS, and the suspension was injected subcutaneously into the backs of the mice, therefore to better demonstrate the discrepancy between the study groups and the control group. Chloroquine (CQ, HY-B1370, MedChemExpress, China) was administered intraperitoneally to the treatment group at a dose of 60mg/kg each day. Tumor volume was measured using calipers and calculated according to the following formula: (length × width^2^) /2. After the 3-week period, subcutaneous tumors were collected and weighed, and the sections were prepared for further use.

### Tissue microarray (TMA) construction

Tissue microarrays were constructed from clinical samples obtained from 149 pairs of PDAC tumor and adjacent normal tissues collected from patients undergoing surgery at Ren Ji Hospital, School of Medicine, Shanghai Jiao Tong University. Written informed consent was obtained from all patients, and the study was approved by the Research Ethics Committee of Ren Ji Hospital (RA-2019-116). Follow-up data were recorded from the date of surgery until cancer-related death.

### Bioinformatic analysis

The raw expression matrix for the single-cell transcriptomic analysis was obtained from the Genome Sequence Archive (GSA) database, accessible at https://bigd.big.ac.cn/gsa, using accession number CRA001160 and GSE212966 25, 26. For example, in CRA001160, a total of 22 PDAC tumor samples (T) and nine control pancreatic samples (N) were collected for further analysis. A list of LMGs was compiled from the "metabolism of lipids" pathway in Reactome. The data was imported into the R (v 4.4.1) package Seurat for further analysis, and each sample was individually filtered to remove cells and genes. Next, mitochondrial, ribosomal, and hemoglobin genes were excluded from the datasets. Ultimately, 33,162 cells and 33,408 genes were retained for further analysis. We applied Harmony to adjust for batch effects across the datasets. The top 2,000 most variable genes were selected using the FindVariableFeatures function in the Seurat package, utilizing the default parameters. Principal component analysis was conducted on the highly variable genes following Z-score normalization. Finally, uniform manifold approximation and projection (UMAP) dimensionality reduction was performed using the top 18 significant principal components (PCs) 27, 28. Clusters were established using the FindClusters function, with a resolution parameter of 0.1. Marker genes for each cluster were identified using the Seurat FindMarkers function, selecting those with a fold change greater than 2. The clusters were annotated to known cell types based on the marker genes using CellMarker 2.0 and PanglaoDB. The expression levels of SLA4A4, ANXA4, and CFTR were utilized to identify ductal cells. Given that tumor cells in PDAC originate from ductal tissue, we initially explored the differences in tumor cells between tumor samples (T) tissues and control pancreatic samples (N) tissues by examining the gene expression profiles within the ductal cell population. To better characterize the malignant state of ductal cell subpopulations, we analyzed the single-cell copy number variation (CNV) profiles in ductal cells using inferCNV, with ductal cells in control pancreatic samples (N) serving as the reference.

### Immunohistochemistry of paraffin-embedded tissues (IHC-P)

Immunohistochemistry for paraffin-embedded sections (IHC-P) was performed as previously described. For IHC-P analysis of xenograft tumors and pancreatic tissues from KPC and KC mice generated in our laboratory, specimens were fixed in 4% PFS, embedded in paraffin, and subjected to IHC staining. Tumor tissues were evaluated based on the intensity of cytoplasmic staining (0 = no staining, 1 = weak staining, 2 = moderate staining, and 3 = strong staining) of OSBPL10 and the percentage of stained area (0 = 0-5%, 1 = 6-35%, 2 = 36-70%, and 3 = 71-100%). Final scores were calculated by multiplying these two values, with the following classifications: “negative” for a score of 0-1, “weak” for a score of 2-4, “moderate” for a score of 4-6, and “positive” for a score of 7-9 29. The scores were collected for the analysis of univariate, multivariate and Kaplan-Meier analyses etc. to better evaluate the effect of *OSBPL10* expression on clinical features and patient lesions.

### Quantitative real-time polymerase chain reaction (qRT-PCR)

Total RNA was extracted from cells using Trizol reagent (Sharebio, Shanghai) according to the manufacturer's instructions. The cDNA synthesis was carried out with the PrimeScript RT Reagent Kit (Takara, Japan), following the provided protocol. qRT-PCR was performed using SYBR Premix Ex Taq (Sharebio, Shanghai) on a 7500 Real-Time PCR system (Applied Biosystems, USA) under the recommended thermal cycling conditions. The relative mRNA expression levels were determined using the 2^-ΔΔCt^ method and normalized to 18S RNA levels. The primer sequences for the target genes are listed in Supplementary Table S1.

### Immunoblotting

Total protein was extracted from cells using RIPA Lysis and Extraction Buffer (89901, Thermo Fisher Scientific, USA) and quantified with a bicinchoninic acid (BCA) Protein Assay Kit (SB-WB013, ShareBio, China). The extracted proteins were separated by sodium dodecyl sulfate-polyacrylamide gel electrophoresis (SDS-PAGE, 20315ES20, YEASEN, China) and transferred onto nitrocellulose membranes. The membranes were blocked with 2% FBS and then incubated overnight at 4 °C with primary antibodies (Supplementary Table S2). Afterward, the membranes were incubated with species-specific secondary antibodies (Supplementary Table S2) for 1 hour at room temperature. Immunoreactive bands were detected using chemiluminescence (BioRad Laboratories, CA, USA).

### Small interfering RNA (siRNA) and cDNA transduction

For the transduction with small interfering RNA (siRNA), Lipofectamine RNAiMAX (#13778150, Thermo Scientific, USA) was used to transiently transfect PDAC cell lines with siRNA and cDNA achieve specific gene knockdown and expression rescue. After 48 hours of transfection, the PDAC cells were collected for qRT-PCR or immunoblotting to evaluate transduction efficiency or for additional assays using the jetPRIME transfection reagent (Polyplus, France). The sequences of cDNA of ATG2A are provided in Supplementary Information 2. The sequences of siRNAs utilized in this study are as follows: si-*ATG2A*-1, sense, 5'-GACACUGCGGUUGCACAAA(dT)(dT)-3', antisense, 5'-UUUGUGCAACCGCAGUGUC(dT)(dT)-3', and si-*ATG2A*-2, sense, 5'-CCUUCACUCUCUCCAGCAA(dT)(dT)-3', antisense, 5'-UUGCUGGAGAGAGUGAAGG(dT)(dT)-3', si-*ATG2A*-3, sense, 5'-GCGAGAUUAUGUCUGUGUU(dT)(dT)-3', antisense, 5'-AACACAGACAUAAUCUCGC(dT)(dT)-3'.

### Gene knockdown and overexpression assays

Lentiviral vectors for the overexpression of *OSBPL10* and the knockdown of *Osbpl10* were acquired from OmicsLink^TM^ Expression Clone (EX-Y4308-Lv137; GeneCopoeia, Inc., Rockville, MD). The vector used for *Osbpl10* knockdown assays was WPE-3'LTR-pUC-Amp-5'LTR-RRE-cPPT-U6-shRNA-SV40-Luc2-IRES-Puro. The vector used for *OSBPL10* knockdown and overexpression assays was WPE-3'LTR-pUC-Amp-5'LTR-RRE-cPPT-CMV-ORF-3xflag-SV40-IRES-Puro. All the sequences mentioned above were described in the Supplementary Information 1 and 2. For the lentiviral transduction assay, cells were planted and cultured in a 6-well plate. After the plantation, lentiviral suspension was added with the presence of 25 × HiTransG P (Genechem, Shanghai, China) for 48 hours, after which the puromycin was added at a density of 2.5μg/ml for stable PDAC cell lines.

### Cell proliferation assays

Cell viability was assessed to demonstrate the proliferation capability of tumor cells, using a Cell Counting Kit-8 (CCK-8, SB-CCK8, Sharebio, China) assay. Cells were seeded into 96-well plates at a density of 2000 cells per well. At days 0, 1, 2, 3, and 4, the cells were incubated with 10 μL of CCK-8 reagent per well for 1 hour, following the manufacturer's instructions. After incubation, absorbance was measured at 450 nm and 600nm using an automatic enzyme-linked immunoassay reader (BioRad Laboratories, CA, USA). Each experiment was conducted in triplicate.

For colony formation assays, cells were plated in six-well plates. After 14 days, the colonies were fixed with 4% PFS and stained with 0.1% crystal violet to demonstrate cell proliferation ability. Photographs were taken, and the colonies were quantified using FIJI 30.

### Immunofluorescence assay

For cell immunofluorescence assays, cells were initially plated with a total number of 1 × 10^6^ each well and cultured in a μ-Slide 8-well (ibidi, Gräfelfing, Germany) for 12 hour. Cells were permeabilized with 0.1% saponin in PBS, blocked with 5% FBS in PBS for 1 hour, and incubated overnight at 4 °C with primary antibodies: OSBPL10 (1:100, Proteintech, China), LAMP1 (1:100, Santa Cruz Biotechnology, USA), and ATG2A (1:100, Santa Cruz Biotechnology, USA). After washing, cells were incubated with secondary antibodies for 1 hour at room temperature. Nuclei were stained with DAPI (1:100, Merck Millipore, USA). Lipid droplets (LDs) were stained with BODIPY (1:1000, Beyotime, Shanghai, China).

For tissue immunofluorescence, freshly collected PDAC samples from Ren Ji Hospital, Shanghai Jiao Tong University, were sectioned into 10 µm slices. Sections were fixed with 4% PFA, subjected to heat-mediated antigen retrieval in pH 6.0 sodium citrate buffer, and blocked with 10% BSA in PBS. Primary antibodies were incubated overnight at 4 °C, followed by incubation with fluorescence-conjugated secondary antibodies. Nuclei were stained with DAPI, and immunofluorescence signals were visualized using confocal microscopy (Leica, Germany).

Quantification of BODIPY (intracellularly) and PLIN2 (in tumor tissue sections) was quantified using FIJI, with colocalization levels scored from 0 to 100. High lipophagy was defined as the top 10% of scores, and low lipophagy as the bottom 10%.

### Transmission electron microscope (TEM)

For TEM, cells (1 × 10⁶) and fresh tissues were collected and fixed in electron microscopy fixative at 4 °C for 2-4 hours. Samples were washed in 0.1M phosphate buffer, followed, then embedded 1% agarose. After fixation with osmium tetroxide, samples were sequentially dehydrated in ethanol, infiltrated with acetone and embedding medium, and polymerized at 60°C for 48 hours. Thin sections (60-80 nm) were cut using an ultramicrotome and mounted on copper grids. Grids were stained with 2% uranyl acetate and 2.6% lead citrate, and analyzed under a transmission electron microscope.

### RNA-sequencing

RNA was extracted from SW1990 cells with OSBPL10 knockdown and the corresponding negative control using RNAiso Plus reagent (Takara Bio, Japan). RNA quality was assessed using a Bioanalyzer (Agilent) and quantified with a NanoDrop spectrophotometer (Thermo Scientific, USA). Only high-quality RNA samples (OD260/280 = 1.8-2.2, RIN ≥ 6.5) were selected for sequencing. RNA sequencing libraries were constructed using the Illumina® Stranded mRNA Prep, Ligation method (Illumina, USA) and sequenced on a NovaSeq 6000 sequencer.

### Proteomic analysis

We utilized lysates from Patu8988 cell with exogenous *OSBPL10* overexpression from immunoprecipitation for an isobaric tag for relative and absolute quantitation (iTRAQ) proteomic experiment. The samples underwent tissue lysis, protein extraction, BCA quantification, SDS-PAGE electrophoresis, Coomassie Brilliant Blue staining, and enzymatic digestion (300 μg per sample) for preparation. Peptides were labeled with TMT reagents according to the manufacturer's protocol (Thermo Scientific, USA). The fractions were dried and prepared for nano-LC-MS/MS analysis. Peptides from each group were mixed in equal amounts and separated using the Pierce™ High-pH Reversed-Phase Peptide Fractionation Kit (Thermo Scientific, USA). The samples were then combined into 10 fractions, which were dried and redissolved in 0.1% formic acid (FA) for mass spectrometry analysis. Peptides from each sample were subjected to chromatographic separation using the Easy nLC 1200 system (Thermo Scientific, USA) and analyzed by data-dependent acquisition (DDA) mass spectrometry on a Q-Exactive HF-X mass spectrometer (Thermo Scientific, USA). The resulting LC-MS/MS raw files were processed using the Sequest HT search engine in Proteome Discoverer software (Version 2.4, Thermo Scientific, USA) to perform a database search. The UniProt-Homo sapiens (Human) [9606]-202249-210712 database was used for the search, and a false discovery rate (FDR) of ≤ 0.01 was applied for filtering and exclusion.

### Co-immunoprecipitation

Cells from two 10cm plates were cultured and harvested using IP lysis buffer containing protease/phosphatase inhibitors. The cell lysates were then sonicated and centrifuged at 4°C. Anti-Flag beads (Selleck, USA) were washed three times with PBST and subsequently incubated with pre-cleared cell lysates at room temperature for 2 hours. The mixture was then washed four times with PBST and eluted with 50 μL of freshly prepared 1× loading buffer, which was heated at 90 °C for 10 minutes before further use. The co-IP experiments were performed to demonstrate the interactions between OSBPL10 and VAPA as well as VAPB.

### Untargeted lipidomic liquid-chromatograph mass spectrometry

Lipidomic analysis was conducted to assess metabolic alterations associated with OSBPL10 knockdown. Lipid extraction was performed using a MeOH/MTBE extraction protocol, followed by LC-MS analysis using an ACQUITY™ Premier CSH C18 column. Data were collected in both positive and negative ion modes on a Thermo QE HF-X mass spectrometer.

### Statistical analysis

Quantitative data are presented as means ± SEM. Statistical analyses were conducted using SPSS 26.0. Cumulative survival times were calculated using the Kaplan-Meier method and analyzed with the log-rank test. The correlation between *OSBPL10* expression and clinical baseline information of PDAC patients in the Ren Ji Cohort was assessed using the χ2 test or Fisher's exact test. Univariate and multivariate Cox regression analyses were performed using the Cox proportional hazards model to identify significant factors affecting patient survival. Continuous variables were compared using a two-tailed Student's t-test or one-way ANOVA. A p-value of < 0.05 was considered statistically significant.

## Results

### Identification of prognosis-related lipophagy in PDAC and the positive correlation between *OSBPL10* and lipophagy

**Figure 1:**
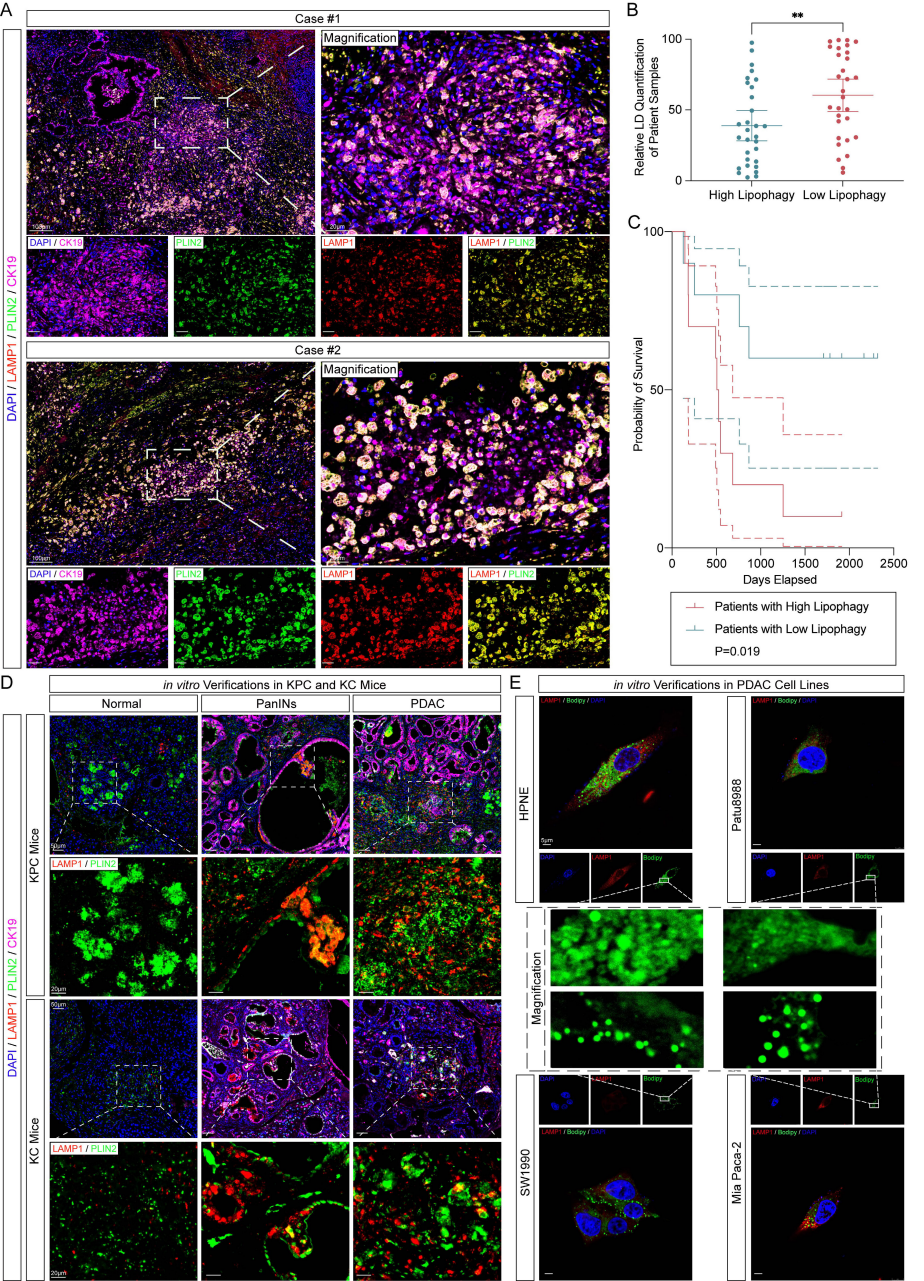
** Identification and verification of lipophagy *in vivo* and *in vitro*.** A, Representative immunofluorescence images of the tumor tissues of PDAC patients in Ren Ji Hospital (n=20, 3 random fields assessed per sample). Scale bar, 100μm and 20μm. Blue, DAPI; Pink, CK19; Green, PLIN2; Red, LAMP1. B, Relative immunofluorescence quantification of PLIN2 in (A) (n=3, 3 random fields assessed per sample, mean ± SEM.; two-tailed unpaired *t* test). C, Kaplan-Meier analysis of 20 patients analyzed in (A) and (B) between high and low lipophagy patients. D, Representative immunofluorescence images of KPC and KC mice about the verification of lipophagy (n=5, 3 random fields assessed per sample). Scale bar, 50μm and 20μm. Blue, DAPI; Green, Plin2; Red, Lamp1. E, Representative immunofluorescence images of HPNE and PDAC cell lines (n=3, 3 random fields assessed per sample). Scale bar, 5μm. Blue, DAPI; Green, PLIN2; Red, LAMP1. (**p<0.01).

To identify lipophagy in PDAC, we first collected tumor tissues from 20 PDAC patients. Paraffin-embedded sections were subjected to immunofluorescence staining for LC3B (an autophagosome marker), LAMP1 (a lysosome marker), and Perilipin 2 (PLIN2, a lipid droplet marker known for its abundant localization on the lipid droplet membrane and high specificity to LDs). Additionally, TEM observation was performed in freshly collected PDAC samples (Supplementary Fig. 1A-B). Patients were categorized into High (n=10) and Low (n = 10) lipophagy groups based on the median value of the quantified lipophagic activity. The extent of lipophagy was assessed by evaluating the colocalization of LAMP1 and PLIN2 as previously reported 11 (Fig. 1A-B). Kaplan-Meier analysis was then performed using clinical follow-up data and overall survival rates. While The Cancer Genome Atlas (TCGA) data showed no significant correlation between PLIN2 and LAMP1 expression and overall survival in PDAC patients, our cohort revealed that patients with high lipophagy activity had significantly worse prognoses (Fig. 1C, Supplementary Fig. 1C-D). To investigate the relationship between lipophagy activity and PDAC progression, immunofluorescence staining was performed on tumor tissues from KPC and KC mice. These results showed that lipophagy was significantly upregulated in pancreatic intraepithelial neoplasia (PanIN) lesions, with the highest levels observed in PDAC regions (Fig. 1D, Supplementary Fig. 2A). Additionally, immunofluorescence staining and immunoblotting on human PDAC cell lines, compared to the normal pancreatic ductal cell line HPNE, confirmed that lipophagy was markedly elevated in PDAC cells (Fig. 1E, Supplementary Fig. 2B-D).

**Figure 2:**
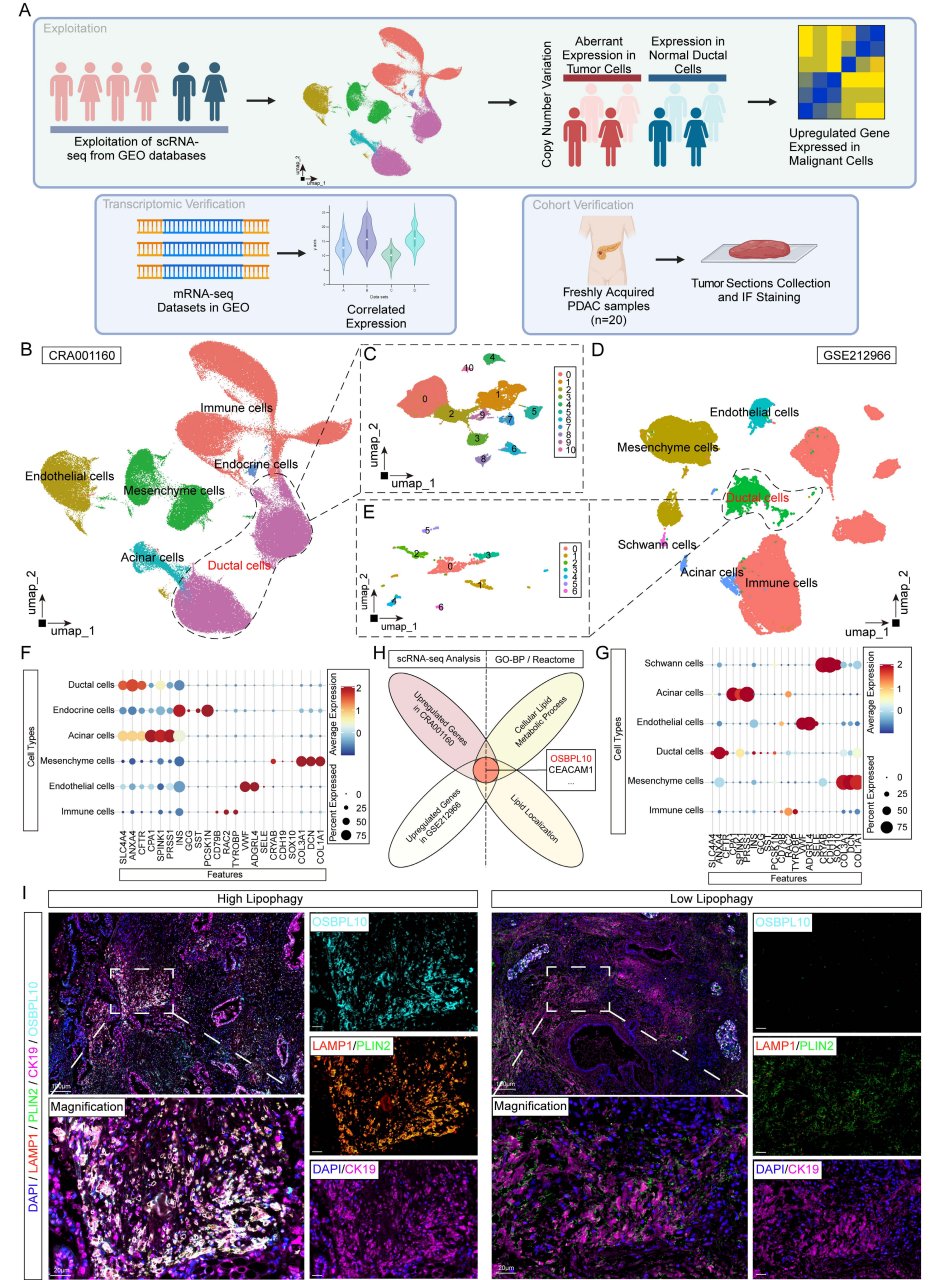
** Single cell RNA sequencing data identification of lipophagy crucial indicator *OSBPL10*.** A, Flow chart demonstrating the sorting and verification process of the potential lipophagy factor. B, UMAP of single cells across PDAC tissues and normal pancreas in CRA001160. C, UMAP of single cells in the ductal cells of CRA001160. D, UMAP of single cells across PDAC tissues and normal pancreas in GSE212966. E, UMAP of single cells in the ductal cells of GSE212966. F, Cell clustering according to the corresponding markers of each cell types in CRA001160. G, Cell clustering according to the corresponding markers of each cell types in GSE212966. H, Venn diagram showing the intersections between the upregulated genes in malignant cells from CRA001160, GSE212966 and corresponding gene sets in Gene Ontology and Reactome. I, Representative immunofluorescence images of the tumor tissues of PDAC patients with high and low lipophagy in Ren Ji Hospital (n=3, 3 random fields assessed per sample). Scale bar, 100μm and 20μm. Blue, DAPI; Pink, CK19; Green, PLIN2; Red, LAMP1; Cyan, OSBPL10.

To further identify factors potentially correlated with lipid metabolism and lipophagy, we performed single-cell transcriptome analysis using publicly available databases (Fig. 2A). After quality filtering and batch effect correction using the Harmony algorithm, we identified six distinct clusters: ductal cells, endothelial cells, acinar cells, mesenchymal cells, immune cells, and endocrine cells. We further re-clustered ductal cells into smaller subsets in both datasets (Fig. 2B-E). In the CRA001160 dataset, cluster 1 was significantly enriched in tumor samples (T) compared to control pancreatic samples (N). To assess the functional roles of these clusters, we analyzed the single-cell copy number variation (CNV) profiles of ductal cells (Fig. 2F, G, Supplementary Fig. 3A-D). Focused analysis on the largest ductal clusters revealed *OSBPL10* as a key gene associated with lipid metabolism, identified by comparing lipid-related gene sets and lipid localization in Reactome and Gene Ontology (Fig. 2H). To validate this finding, we performed immunofluorescence staining on PDAC tissues from patients with the highest and lowest lipophagy activity, showing that *OSBPL10* expression was significantly elevated in regions with high lipophagy (Fig. 2I). Further analysis of *OSBPL10* expression in our single-cell dataset and external datasets from TCGA and Gene Expression Omnibus (GEO) robustly confirmed that *OSBPL10* was upregulated in PDAC tissues and strongly correlated with lipophagy (Supplementary Fig. 3F-G, Supplementary Fig. 4A-B).

Taken together, these results suggest that high lipophagy activity in PDAC is associated with poor prognosis, and *OSBPL10* expression is positively correlated with lipophagy in PDAC.

### High* OSBPL10* expression enhances lipophagy, thereby promoting PDAC cell proliferation *in vitro*

**Figure 3:**
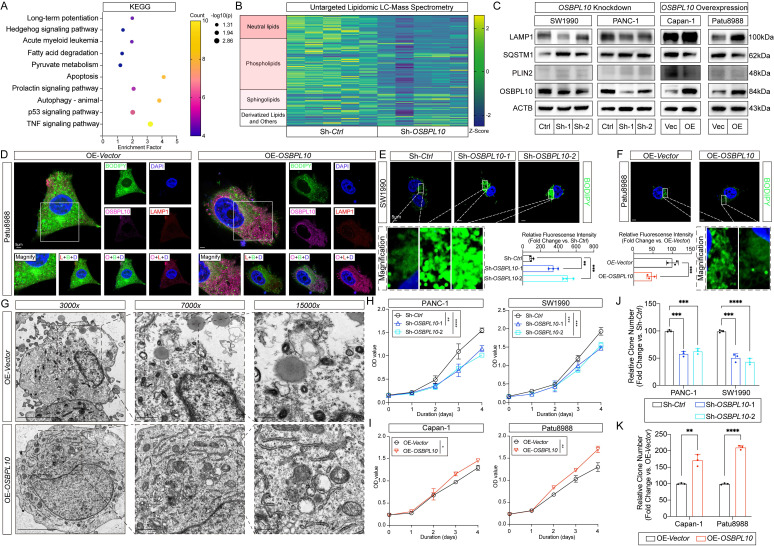
***OSBPL10* Expression positively correlates with lipophagy.** A, KEGG enrichment analysis of the mRNA-seq results between the control group and *OSBPL10^KD^* group. B, Heat map of the lipid LC-MS results between the control group and *OSBPL10^KD^* group. C, Immunoblotting of the corresponding indicated markers of lipophagy in both *OSBPL10^KD^* and *OSBPL10^OE^* cells. D, Representative images of immunofluorescence staining of OSBPL10, LAMP1 and BODIPY in *OSBPL10^OE^* cell line (n=5, 3 random fields assessed per sample). Blue, DAPI; Green, BODIPY; Red, LAMP1. Pink, OSBPL10. Scale bar, 5μm. E-F. Representative images of immunofluorescence staining and quantification of BODIPY in *OSBPL10^KD^* (E) and *OSBPL10^OE^* (F) cell lines (n=3, 3 random fields assessed per sample, mean ± SEM.; two-tailed unpaired *t* test). Blue, DAPI; Green, BODIPY. Scale bar, 5μm. G, Representative transmission electron microscope images of *OSBPL10^OE^* cell line (n=3, 3 random fields assessed per sample), Scale bar, 5μm, 1μm and 0.5μm. H-I, Cell viability of the *OSBPL10^KD^* (H) and *OSBPL10^OE^* (I) cells. (n = 3, mean ± SEM.; one-way repeated-measures ANOVA). J-K, Quantification of colony formation assays of *OSBPL10^KD^* (J) and *OSBPL10^OE^* (K) cells (n=3, mean ± SEM.; two-tailed unpaired *t* test). (*p<0.05, **p<0.01, ***p<0.005, ****p<0.001).

To investigate the role of *OSBPL10* in lipophagy in PDAC cells, we first assessed its expression in HPNE and eight PDAC cell lines. Lentiviral-mediated knockdown and overexpression of *OSBPL10* were performed in selected PDAC cell lines (Supplementary Fig. 6A-B). Based on endogenous expression levels, PANC-1 and SW1990 cells, with high endogenous *OSBPL10* expression, were selected for knockdown, while Capan-1 and Patu8988 cells, with low *OSBPL10* expression, were chosen for overexpression (Supplementary Fig. 6C-F). To validate the role of *OSBPL10* in lipophagy, transcriptomic sequencing was performed on the *OSBPL10* knockdown (*OSBPL10^KD^*) SW1990 cell line. KEGG analysis revealed significant downregulation of pathways related to fatty acid degradation and autophagy, while Gene Ontology (GO) analysis indicated that OSBPL10 was localized to the plasma membrane region (Fig. 3A, Supplementary Fig. 5A-B). Additionally, lipidomic analysis using liquid chromatography-mass spectrometry demonstrated that higher *OSBPL10* expression was associated with an increase in intracellular neutral lipids, particularly triglycerides, indicating a significant increase in lipid metabolic activity (Fig. 3B, Sup. Fig. 5C-D). Elevated *OSBPL10* expression also correlated with upregulation of phospholipids and sphingolipids, providing strong evidence for enhanced intracellular membrane activity (Fig. 3B, Sup. Fig. 5D-E). KEGG analysis further confirmed the enrichment of the autophagy pathway, suggesting that lipophagy was induced by elevated *OSBPL10* expression (Supplementary Fig. 5C-E). To verify these findings, we performed qRT-PCR and immunoblotting on both *OSBPL10^KD^* and *OSBPL10^OE^* cell lines. Consistent with previous results, LAMP1 and SQSTM1 revealed that autophagic activity was upregulated in the *OSBPL10^OE^* cells, while PLIN2 expression demonstrated that lipid droplet level was significantly reduced, confirming the role of *OSBPL10* in promoting lipophagy (Fig. 3C, Supplementary Fig. 6G-H).

Further, we performed immunofluorescence assays on *OSBPL10^KD^* and *OSBPL10^OE^* cells to examine lipophagy activity. In SW1990 cells with *OSBPL10* knockdown, we observed abnormal LD accumulation compared to the control group (Supplementary Fig. 7B-C). In contrast, *OSBPL10* overexpression in Patu8988 cells enhanced lipophagy, as evidenced by significantly increased colocalization of LAMP1 with BODIPY (Fig. 3D-F, Supplementary Fig. 7C). Additionally, TEM images of *OSBPL10^OE^* Patu8988 cells revealed a significant increase in the number of autophagosomes, while lipid droplet accumulation decreased upon *OSBPL10* upregulation (Fig. 3G, Supplementary Fig. 7D). Furthermore, cell proliferation assays demonstrated that high OSBPL10 expression significantly promoted the proliferation of PDAC cells (Fig. 3H-K, Supplementary Fig. 7E).

**Figure 4:**
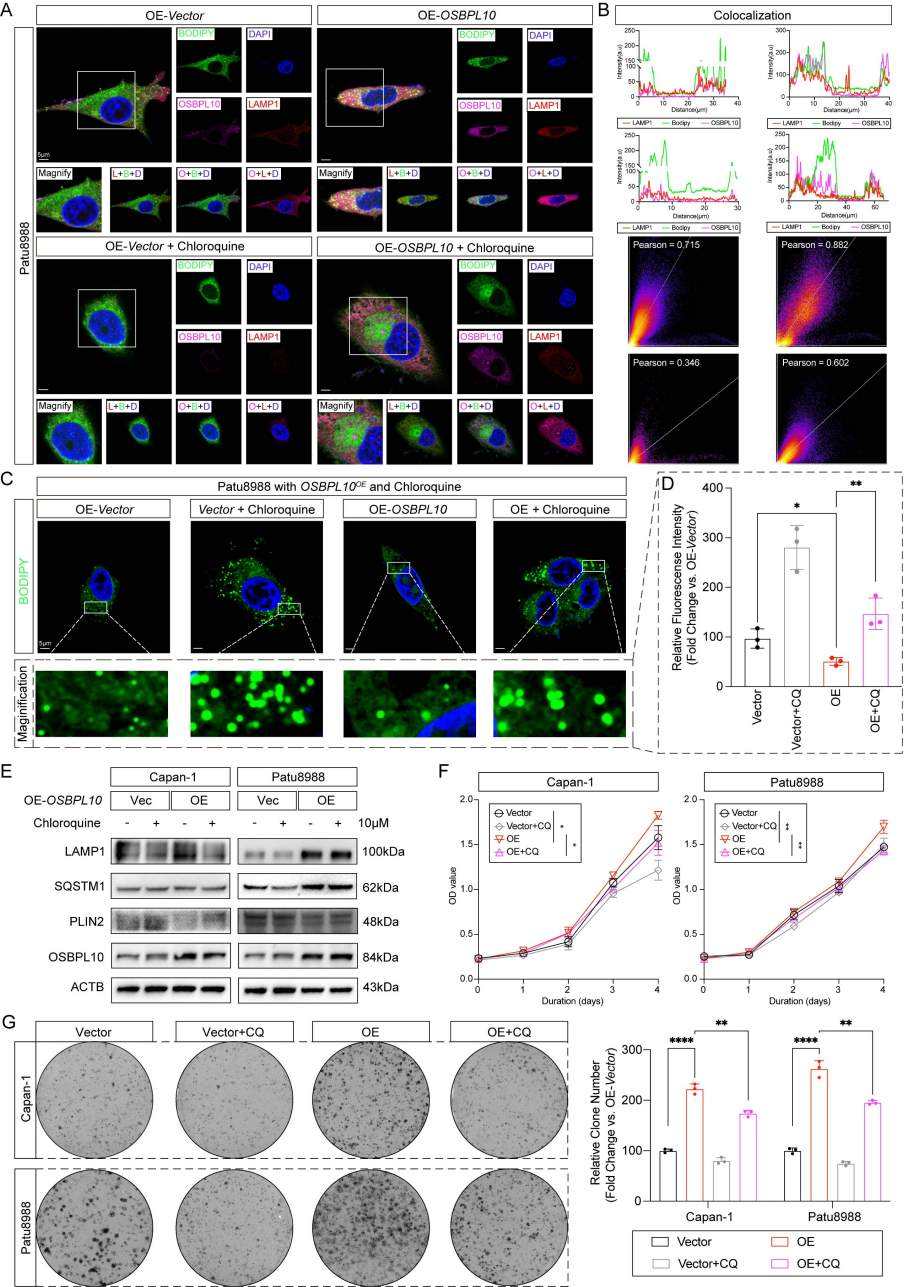
** Upregulated *OSBPL10* expression induced lipophagy could be impeded by chloroquine.** A, Representative images of immunofluorescence staining of OSBPL10, LAMP1 and BODIPY in *OSBPL10^OE^* cell line when treated with 10μM of chloroquine (n=5, 3 random fields assessed per sample). Blue, DAPI; Green, BODIPY; Red, LAMP1. Pink, OSBPL10. Scale bar, 5μm. B, Quantification of the colocalization of LAMP1 and BODIPY, and the correlation analysis between LAMP1 and OSBPL10 in (A). C, Representative images of immunofluorescence staining of BODIPY in *OSBPL10^OE^* cell line when treated with chloroquine (n=3, 3 random fields assessed per sample mean ± SEM.; two-tailed unpaired *t* test). Blue, DAPI; Green, BODIPY. Scale bar, 5μm. D, Relative quantification of BODIPY in *OSBPL10^OE^* cell line when treated with 10μM of chloroquine, n=3, 3 random fields assessed per sample, mean ± SEM.; two-tailed unpaired *t* test. E, Immunoblotting examining corresponding indicated marker of lipophagy in *OSBPL10^OE^* cells when treated with 10μM of chloroquine. F, Cell viability of the *OSBPL10^OE^* cells when treated with 10μM of chloroquine. (n = 3, mean ± SEM., one-way repeated-measures ANOVA). G, Colony formation assays and related quantification of *OSBPL10^OE^* cells when treated with 10μM of chloroquine. (n=3, mean ± SEM.; two-tailed unpaired *t* test). (*p<0.05, **p<0.01, ***p<0.005, ****p<0.001).

To examine whether autophagy is required for these effects, we inhibited lysosomal function with chloroquine (CQ), a well-established autophagy inhibitor. Immunofluorescence assays showed that CQ treatment caused substantial LD accumulation, reduced LAMP1-BODIPY colocalization, and diminished correlation between LAMP1 and OSBPL10 (Fig. 4A-B). Consistent with these findings, the LD content was significantly increased in the CQ-treated cells, confirming that autophagy inhibition impaired lipophagy (Fig. 4C-D). Immunoblotting further confirmed the decrease in lipophagy markers in CQ-treated cells (Fig. 4E). Additionally, cell proliferation assays demonstrated that CQ treatment significantly attenuated the proliferative effect of *OSBPL10* overexpression in both cell lines (Fig. 4F-G).

In summary, these findings suggest that *OSBPL10* expression promotes lipophagy, which in turn enhances cell proliferation. This effect can be impeded by the disruption of autophagosome function, underscoring the importance of lipophagy in PDAC progression.

### *OSBPL10* regulates lipophagy in an ATG2A-dependant manner

**Figure 5:**
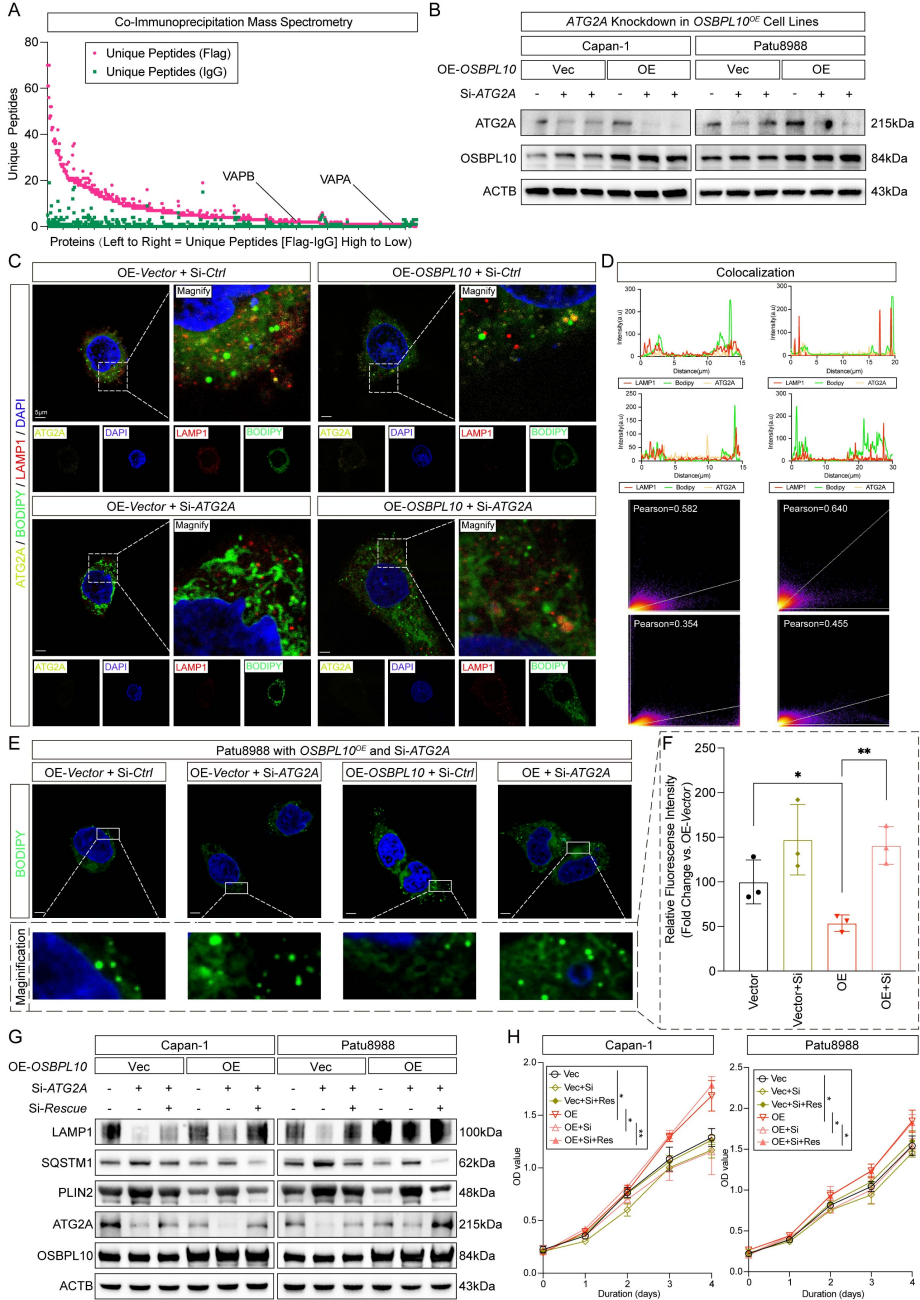
** Promoted lipophagy by *OSBPL10* expression is facilitated by ATG2A-mediated rapid lysosomal repair.** A, The result of the mass spectrometry assay of *OSBPL10^OE^* cells from the samples of co-immunoprecipitation assay. B, Immunoblotting demonstrating the knockdown of si-RNA assays of *ATG2A* in *OSBPL10^OE^* cells. C, Representative images of immunofluorescence staining of ATG2A, LAMP1 and BODIPY in *OSBPL10^OE^* cell line with si-*ATG2A* (n=5, 3 random fields assessed per sample). Blue, DAPI; Green, BODIPY; Red, LAMP1. Yellow, ATG2A*.* Scale bar, 5μm. D, Quantification of the colocalization of LAMP1 and BODIPY, and the correlation analysis between LAMP1 and BODIPY in (C). E. Representative images of immunofluorescence staining of BODIPY in *OSBPL10^OE^* cell line with si-*ATG2A* (n=3, 3 random fields assessed per sample). Blue, DAPI; Green, BODIPY. Scale bar, 5μm. F, Relative quantification of BODIPY in *OSBPL10^OE^* cell line with si-*ATG2A*, n=3, 3 random fields assessed per sample, mean ± SEM.; two-tailed unpaired t test. G, Immunoblotting examining corresponding indicated marker of lipophagy in *OSBPL10^OE^* cells with si-*ATG2A* and *ATG2A*-cDNA (n = 3, mean ± SEM.; one-way repeated-measures ANOVA). H, Cell viability of the *OSBPL10^OE^* cells with si-*ATG2A* and *ATG2A*-cDNA (n = 3, mean ± SEM., one-way repeated-measures ANOVA). (*p<0.05, **p<0.01).

To elucidate the molecular mechanism by which OSBPL10 promotes lipophagy, we performed mass spectrometry to identify its interacting proteins. This analysis revealed associations with VAPA and VAPB, two proteins previously reported to cooperate functionally with OSBPL10 (Fig. 5A). These interactions were further validated by immunoblotting and were consistent with predictions from the BioGRID and STRING databases (Supplementary Fig. 8A-B). As described in the work of Tan *et al.*, VAPA and VAPB both interacts with members of the OSBP family, including OSBPL10, in order to have the damaged lysosome closer to the ER, therefore proceed the repairing system with the primary involvement of PI4K2A and ATG2A 21. Given that several genes related to lysosomal functions showed altered expression on account of *OSBPL10* knockdown, as well as previous studies by Tan *et al.* have reported the unique functions that OSBPL10 plays in rapid lysosomal repair, we speculated whether OSBPL10 was involved in the regulation of the lipophagy process, therefore affecting PDAC cell proliferation 21. First, we verified the study of Tan et.al by using the wild type PDAC cells of Patu8988 and Mia Paca-2 to observe the correlation between OSBPL10 and ATG2A (Supplementary Fig. 8C). As previously reported to be related to intracellular LD morphology and distribution, *ATG2A*, the effective gene in *ATG2* family which was pivotal to rapid lysosomal repair machinery, is known to be associated to the mediating effect of isolation membrane and the ER, moreover, *ATG2A* facilitates autophagosome assembly, while binding to the ER exit site, the membrane source for autophagosome 31-34. Hence, according to the former study, we wished to further speculate whether *OSBPL10* promote lipophagy by ATG2A and its participation in rapid lysosomal repair 21 (Fig. 5B). Later, we performed immunofluorescence assays on the *OSBPL10^OE^* cell lines as well as those with *ATG2A* knockdown, and we observed an accumulation of LDs, and with *ATG2A^KD^*, the colocalization between lysosomes and LDs also showed a reduction, while the lysosome function was barely affected, suggesting that low *ATG2A* expression significantly impeded elevated lipophagy by high *OSBPL10* expression (Fig. 5C-F). For more evidence, immunoblotting and cell proliferation assays were performed and also displayed that lysosome damage and lipophagy were indeed deterred by *ATG2A* knockdown, while the restoration of *ATG2A* knockdown have considerably reversed the impairment of rapid lysosomal repair and cell growth, without affecting PI4K2A recruitment 21 (Fig. 5G-H, Supplementary Fig. 8D-E).

Together, these results indicates that the effect of *OSBPL10* on lipophagy was modulated by ATG2A-mediated rapid lysosomal repair, which regulated the function of lysosomes, therefore posing an impact on lipophagy.

### Tumor growth and intracellular lipophagy are facilitated by *OSBPL10* and can be dampened by chloroquine

**Figure 6:**
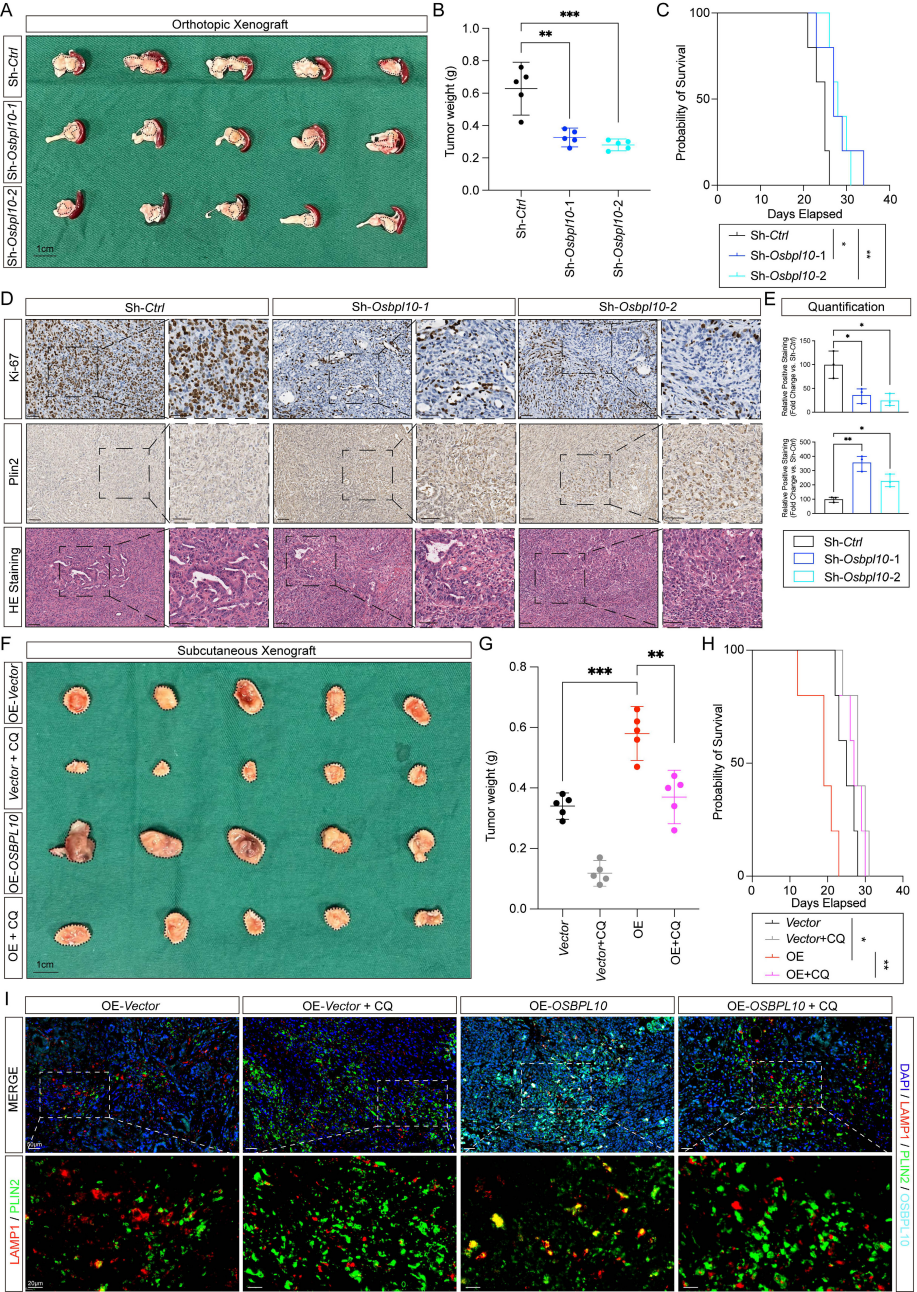
** Tumor growth and intracellular lipophagy are facilitated by *OSBPL10* and can be dampened by CQ.** A. Images taken when PDAC orthotopic xenografts were retrieved from mice. B. Tumor weight in the control group and *Osbpl10^KD^* groups. C. Kaplan-Meier analysis of overall survival in the control group and *Osbpl10^KD^* groups. D. Representative immunohistochemical staining, HE staining images of tumors from the control group and *Osbpl10^KD^* groups with Ki-67 and Plin2 (n=3, 3 fields assessed per sample). Scale bar, 50μm and 20μm. E. Corresponding H-scores of immunohistochemical stainings from (D) (n=3, 3 random fields assessed per sample, mean ± SEM.; two-tailed unpaired *t* test). F. Images taken when subcutaneous xenografts were retrieved from mice. G. Tumor weight in the control group,* OSBPL10^OE^* group and groups treated with CQ. H, Kaplan-Meier analysis of overall survival in the control group, *OSBPL10^OE^* group, and groups treated with CQ. I, Representative immunofluorescence assays from the control group, *OSBPL10^OE^* group, and groups treated with CQ. (n=3, 3 random fields assessed per sample, mean ± SEM.; two-tailed unpaired *t* test). Scale bar, 50μm and 20μm. (*p<0.05, **p<0.01, ***p<0.005).

To further investigate the *in vivo* role of *OSBPL10* in tumor growth and lipophagy, we utilized orthotopic and subcutaneous xenograft models. *Osbpl10* expression was knocked down in the KPC1199*^luc^* cell line for the orthotopic xenograft model (Fig. 6A-B, Supplementary Fig. 9A). Upon resection, pancreatic tumors were weighed, and tumors from the knockdown group were significantly lighter compared to those from the control group. Mice injected with *Osbpl10* knockdown (*Osbpl10^KD^*) cells also showed worse overall survival (Fig. 6C). *In vivo* imaging was used to track tumor growth over time, and the results showed slower tumor progression in the knockdown group compared to the control group (Supplementary Fig. 9B-C). Immunohistochemical analysis of excised tumors showed significantly lower Ki-67 staining and elevated caspase-3 expression in the knockdown group, indicating reduced proliferation and increased apoptosis (Fig. 6D-E, Supplementary Fig. 9D). Additionally, lipophagy markers, as Plin2 and Lamp1, were evaluated, and knockdown of *Osbpl10* resulted in a marked reduction in lipophagy activity (Fig. 6F, Supplementary Fig. 9D).

To further explore the effect of OSBPL10 and intracellular lipophagy on PDAC progression, we constructed subcutaneous xenograft models using the *OSBPL10^OE^* Patu8988 cell line, with mice receiving intraperitoneal CQ treatment each day at the dosage of 60mg/kg 35, 36. As expected, overexpression of *OSBPL10* led to increased tumor weights, consistent with previous results, and CQ treatment alleviated tumor growth in both control and *OSBPL10^OE^* groups. Mice implanted with *OSBPL10^OE^* cells showed significant reductions in tumor weights, volumes, and overall survival following CQ treatment (Fig. 6F-H, Supplementary Fig. 9E). To assess lipophagy levels, tumor samples were subjected to immunofluorescence assays. As expected, analysis of the colocalization of LAMP1 and PLIN2 revealed significantly elevated lipophagy in tumors with high *OSBPL10* expression. In contrast, lipophagy was notably reduced in tumors treated with CQ (Fig. 6I).

In conclusion, these findings demonstrate a positive correlation between *OSBPL10* expression and tumor growth *in vivo*. They also confirm the critical role of *OSBPL10* in regulating lipophagy in PDAC tumor cells. Importantly, CQ treatment, particularly in tumors with high *OSBPL10* expression, mitigates tumor growth through the inhibition of lipophagy.

### High *OSBPL10* expression indicates poor prognosis in PDAC patients

**Figure 7:**
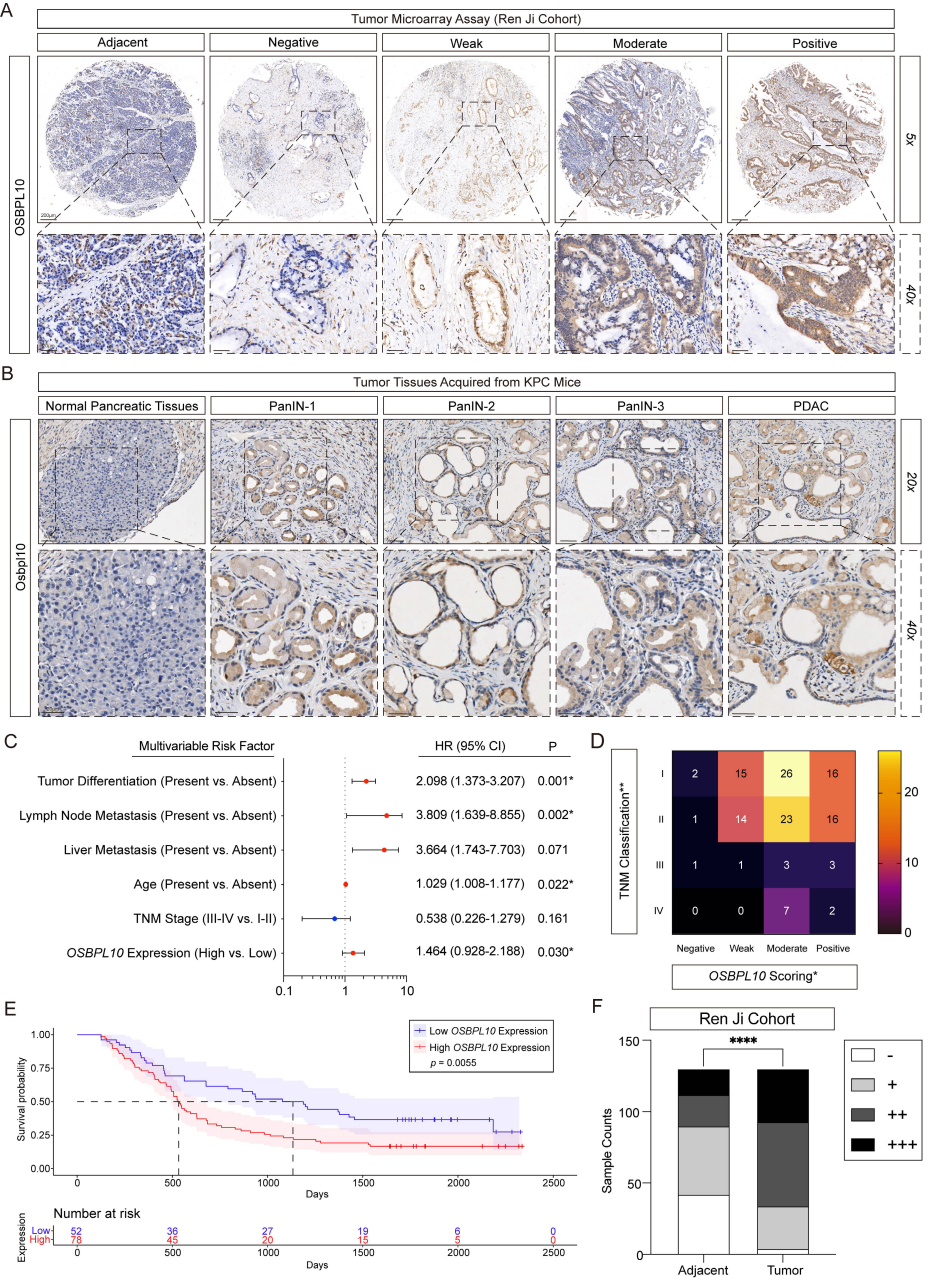
***OSBPL10***** expression is elevated in PDAC and high *OSBPL10* expression indicates poor clinical outcome in PDAC patients.** A, Representative immunohistochemical scoring images of* OSBPL10* expression in 130 pancreatic ductal adenocarcinoma adjacent tissues and matching tumor tissues in the Renji TMA (n=130, 3 fields assessed per sample). Scale bar, 200 μm, and 20 μm. B, Representative immunohistochemical staining images of *Osbpl10* expression in KPC mice at different stages of PDAC progression (n=5 samples, 3 fields assessed per sample). PanIN, pancreatic intraepithelial neoplasms. Scale bar, 50 μm and 20 μm. C, Multivariate Cox regression analysis of clinicopathological factors in the Ren Ji TMA. CI, confidence interval. D. Heatmap of *OSBPL10* expression distribution in the 4 levels of TNM classification based on the immunohistochemical scoring of the Ren Ji TMA, two-way ANOVA. E, Kaplan-Meier analysis of the correlation between the overall survival rate and the expression of *OSBPL10* according to the Ren Ji cohort. F, OSBPL10 staining H-score of adjacent and the matched tumor tissues, two-way ANOVA. Negative (-), weak (+), moderate (++) or positive (+++). (****p<0.001).

To comprehensively evaluate the expression pattern and clinical relevance of *OSBPL10*, we first analyzed TCGA data and found that *OSBPL10* mRNA levels were upregulated in several malignancies, including ovarian cancer (OV) and acute myeloid leukemia (LAML) (Supplementary Fig. 10A). To further assess its clinical significance in PDAC, we constructed a tissue microarray (TMA) containing tumor and adjacent tissues from 149 patients who underwent surgery at Ren Ji Hospital, Shanghai Jiao Tong University School of Medicine. Intact clinicopathological features were collected were available for 130 patients, and clinical follow-up data for 112 patients. IHC staining of the TMA was evaluated by two independent pathologists, who scored both the distribution and intensity of *OSBPL10* expression (Fig. 7A).

In parallel, IHC was performed on pancreatic tissues from KPC and KC mice to investigate *OSBPL10* expression during PDAC development. The results showed progressively elevated *OSBPL10* expression in PanIN and PDAC lesions (Fig. 7B, Supplementary Fig. 10B-C). Analysis of the TMA revealed that *OSBPL10* served as an independent clinicopathological indicator in our cohort and was positively correlated with TNM stage, tumor size, and tumor differentiation (Fig. 7C-D, Supplementary Fig. 10D). Furthermore, survival analysis demonstrated that patients with high *OSBPL10* expression had significantly poorer overall survival compared to those with low expression (Fig. 7E). Quantitative comparison confirmed that *OSBPL10* expression levels were markedly higher in tumor tissues than in adjacent normal tissues (Fig. 7F). Consistently, analysis of the TCGA-PAAD dataset showed that patients with elevated *OSBPL10* expression had reduced recurrence-free survival and overall survival, supporting its association with poor prognosis (Supplementary Fig. 11A-B).

In summary, these results indicate that *OSBPL10*, as a key lipophagy factor, is significantly upregulated in human PDAC tissues and serves as a predictor of poor clinical outcomes.

## Discussion

In this study, we demonstrated the presence and significance of lipophagy in PDAC, highlighting its role as an effective mechanism for tumor cells to mobilize lipid reserves stored in LDs. Reprogramming of lipid metabolism, particularly fatty acid utilization, is a well-established hallmark of PDAC. Our study extends this concept by uncovering the role of lipophagy in mobilizing lipid droplets 10, 37. Epidemiological studies have consistently identified obesity as a major risk factor for cancer progression, and in PDAC, fatty acid utilization and consumption have emerged as a critical area of study 19, 38, 39. Even under conditions of extracellular fatty acid abundance, cancer cells exploit all available fatty acids to sustain growth 40. In early studies, researchers have demonstrated multiple genes with aberrant upregulation in solid tumors including hepatocellular carcinoma, prostate cancer, colorectal cancer, and PDAC 6, 38, 41. LDs, commonly described as “lipid reservoirs,” play a silent yet essential role in cancer metabolism. Neutral lipids stored in LDs, primarily triacylglycerols, are tightly regulated to maintain cellular homeostasis 42. LDs may also be synthesized *de novo* under stress conditions such as nutrient deprivation 43. Within the complex tumor microenvironment of PDAC, nutrient availability is often limited, and LDs provide a critical energy source under these conditions. Despite the central importance of LDs, relatively few studies have focused on the mechanisms of LD breakdown, which is necessary for the release of free fatty acids for metabolic use.

Macroautophagy, generally referred to as autophagy, plays a central role in cellular recycling and degradation of intracellular components 44. While autophagy has context-dependent pro- and anti-tumor functions, our data specifically highlight lipophagy as a critical adaptation supporting PDAC progression, filling a gap in current knowledge 22, 45-47. Accumulating evidence supports a tumor-promoting role for autophagy, based on the upregulation of autophagy-related genes and proteins in various cancers 22, 48. Lipophagy, a selective form of autophagy, was first identified in 2009 in hepatocytes and has since provided new insights into the relationship between intracellular lipid metabolism and cellular physiology 49. PDAC cells, however, exhibit highly unorthodox lipid metabolism, raising the question of how they sustain rapid progression in nutrient-deprived tumor microenvironments 50, 51. Our study positions enhanced lipophagy not merely as a correlative event but as a critical adaptive metabolic mechanism that actively fuels PDAC progression, offering a novel perspective on the metabolic adaptations of PDAC cells.

In our study, we identify *OSBPL10* as a key factor of lipophagy in PDAC. This biological process ensures a continuous supply of free fatty acids regardless of external nutrient fluctuations. These fatty acids serve dual purposes: they fuel ATP production via β-oxidation and act as essential building blocks for the synthesis of proliferating cellular membranes and signaling lipid mediators 39. *OSBPL10*-enhanced lipophagy directly supports tumor growth by sustaining pro-proliferative signaling and biosynthesis. The products of lipophagy, fatty acids and cholesterol, are not merely metabolic fuel. Free fatty acids can act as ligands for peroxisome proliferator-activated receptors, activating transcriptional programs linked to proliferation and metastasis 52. Moreover, efficiently recycled cholesterol is vital for maintaining membrane integrity and cholesterol-rich lipid rafts, which are essential platforms for the signaling of key growth-promoting receptors, including those in the KRAS pathway, a nearly universal driver in PDAC 53. While previous studies have established *OSBPL10* as an oncogene promoting PDAC progression through the VEGF/AKT pathway, and bioinformatic analyses have corroborated its prognostic significance, its specific role in metabolic reprogramming remained unexplored 54, 55. The *OSBPL10*-lipophagy axis is also distinct from several established mechanisms in the field. Unlike *OSBPL8*, which was identified as a direct lipophagy receptor that bridges lipid droplets to autophagosomes to mediate their degradation in hepatocytes, *OSBPL10* operates upstream as a guardian of lysosomal integrity via ATG2A-mediated repair. While recent work highlights that glycolysis-suppressed PDAC cells become dependent on lipophagy for survival, our discovery of the *OSBPL10*-lipophagy axis provides the mechanistic explanation for how this critical process is specifically upregulated in tumors 8, 37. Our work bridges this gap by discovering an unprecedented mechanism: *OSBPL10* drives PDAC progression by specifically enhancing lipophagy through ATG2A-mediated rapid lysosomal repair. This positions *OSBPL10* not merely as a general oncogene but as a critical metabolic sensor and facilitator. It directly regulates the integrity of the degradative compartment, lysosomes, thereby ensuring efficient lipid droplet turnover to fuel tumor growth. This ATG2A-dependent lysosomal repair mechanism represents a previously unrecognized layer of lipophagy regulation in cancers.

To date, autophagy and its related pathways have been closely linked to cancer cell progression and survival, highlighting autophagy as a potential therapeutic target 9, 47, 56, 57. However, few therapeutic targets and inhibitors have been identified, and the direct crosstalk between selective autophagy and intracellular metabolism remains underexplored. Our findings nominate *OSBPL10* as a candidate therapeutic target in PDAC. Direct inhibition of *OSBPL10* faces challenges including the lack of existing inhibitors and potential on-target toxicities in normal tissues with high lipid turnover 54. In contrast, repurposing lysosomotropic agents like CQ or Bafilomycin A1 offers a more immediate translational avenue, as evidenced by our study and ongoing oncology trials evaluating its combination with gemcitabine and other agents in PDAC 58. Although broad autophagy blockade may limit its therapeutic effects, its rational combination with standard therapies currently presents the most viable strategy to exploit the *OSBPL10*-mediated lipophagy dependency in PDAC 58. Meanwhile, we observed heterogeneity in lipophagic activity among not only among cancer cells, but also among other components in the tumor microenvironment, and we wish to conduct further investigation in future studies on carcinogenesis in PDAC and other malignant tumors. Moreover, further proof of direct interaction between OSBPL10 and VAPA/VAPB awaits further assays to ensure the mechanism of rapid lysosomal repair. Moreover, by comparing other in-depth published works about lipophagy, including its perturbation towards intracellular lipid metabolism via SREBF1, or its specific receptor, ATG14, that has been recently discovered, more work needs to be done in order to depict an elaborate atlas of lipophagy not only in PDAC, but also in more diseases 59, 60.

By uncovering the mechanism of lipophagy regulation in PDAC, our study provides new insights into the role of intracellular fatty acid metabolism and energy utilization, and lays a foundation for future investigations into therapeutic strategies targeting lipophagy.

## Supplementary Material

Supplementary figures and tables.

## Figures and Tables

**Figure 8:**
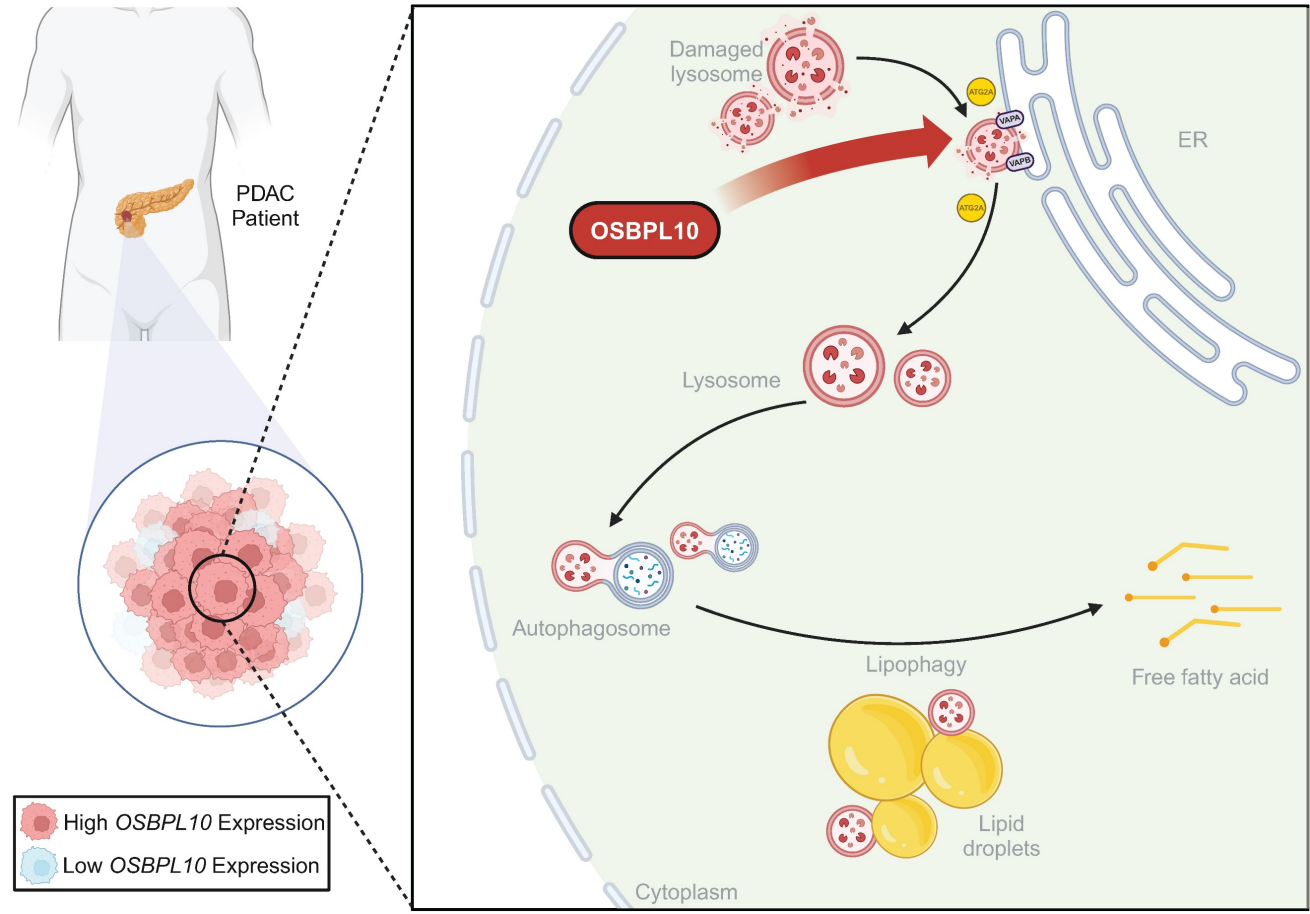
** Graphical abstract of high *OSBPL10* expression facilitates lipophagy via ATG2A-mediated rapid lysosomal repair to support PDAC proliferation**.

## Data Availability

The data are available within the Article, Supplementary Information, or available from the authors upon request. Source data are provided with this paper.
