# Supplementary material for: OSBPL10 Drives Lipophagy-Mediated Lipid Mobilization to Promote Pancreatic Ductal Adenocarcinoma Progression
Source: Int J Biol Sci. 2026 Mar 25;22(7):3769–87. doi: 10.7150/ijbs.125552 (PMC13086084; doi:10.7150/ijbs.125552)
Supplement: Supplementary file 1 — Supplementary figures and tables. [file ijbsv22p3769s1.pdf]

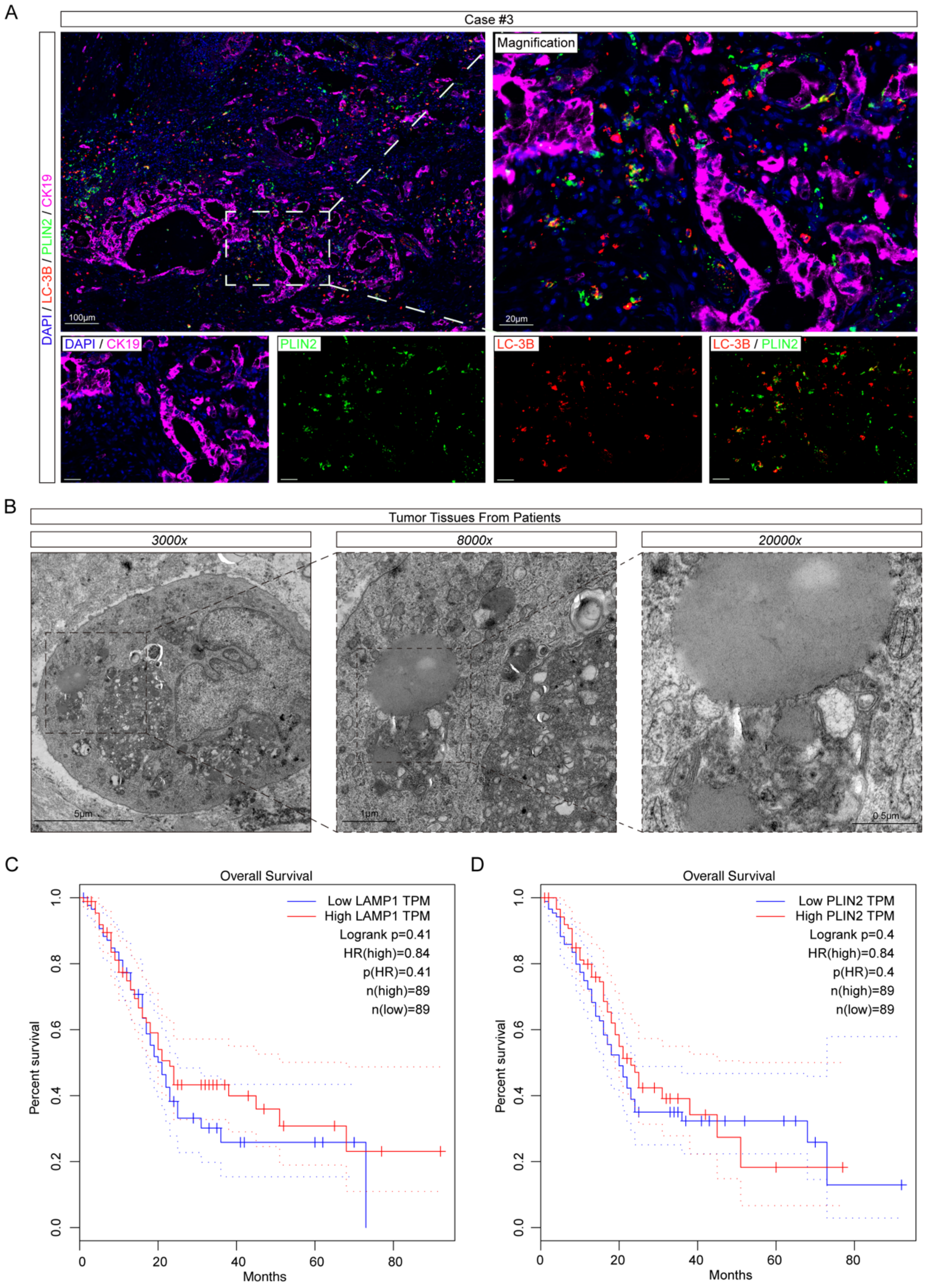

Supplementary Figure. 1 Expression profiles and prognostic value of lipophagy-related markers in pancreatic ductal adenocarcinoma.

A, Representative immunofluorescence images of the tumor tissues of PDAC patients in Ren Ji Hospital (n=3, 3 random fields assessed per sample). Scale bar, 100μm and 20μm. Blue, DAPI; Pink, CK19; Green, PLIN2; Red, LC-3B.

B, Representative transmission electron microscope images of *OSBPL10*^{OE} cell line (n=3, 3 random fields assessed per sample), Scale bar, 5μm, 1μm and 0.5μm.

C-D, Kaplan-Meier analysis of LAMP1 (C) and PLIN2 (D) in TCGA-PAAD database.

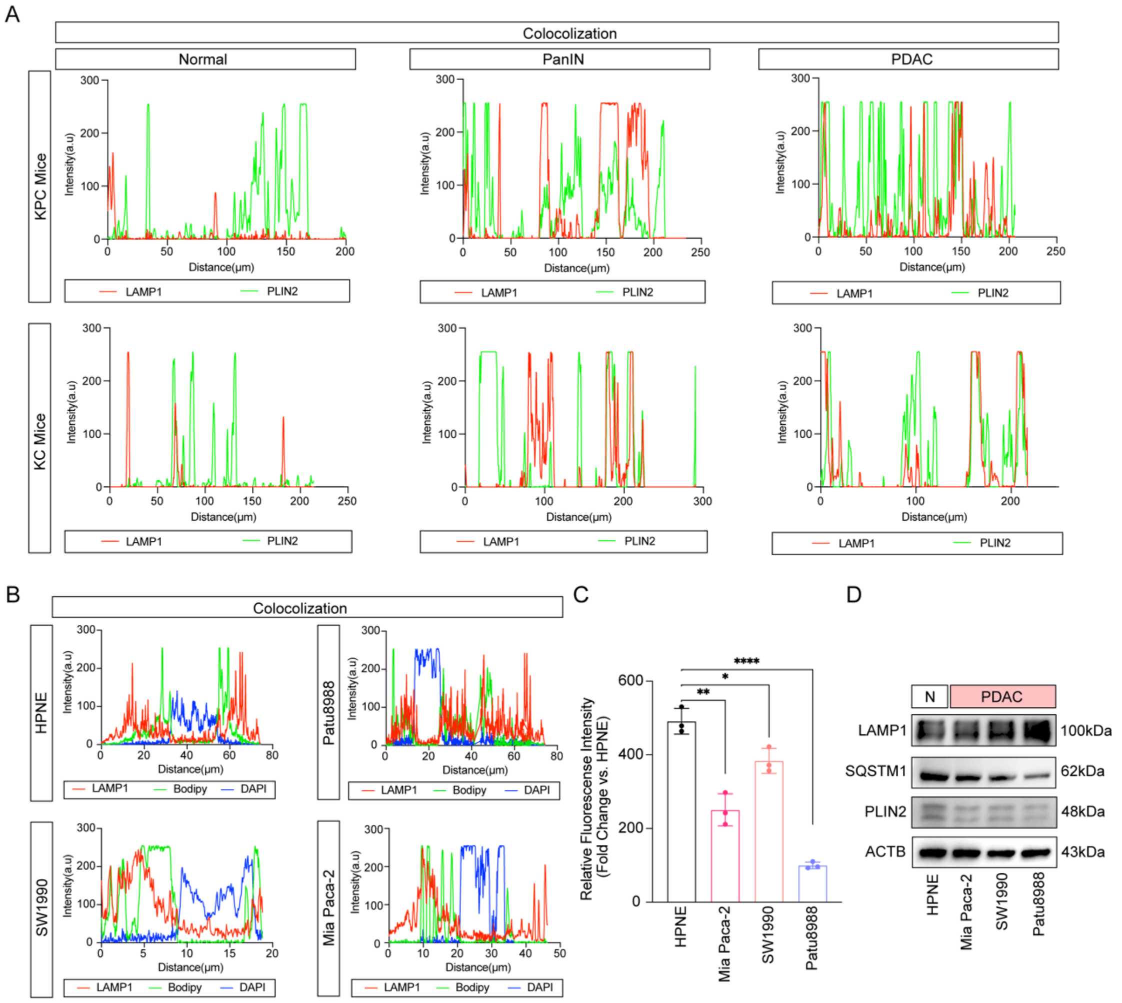

Supplementary Figure. 2 *In vivo* and *in vitro* quantitative analysis of enhanced lipophagy in pancreatic ductal adenocarcinoma.

A, Corresponding colocalizations of Fig. 1D.

B, Corresponding colocalizations of Fig. 1E.

C, Corresponding quantification of BODIPY intensity between PDAC cell lines and HPNE cell line (n=3, 3 random fields assessed per sample, mean ± SEM.; two-tailed unpaired *t* test).

D, Immunoblotting of lipophagy-related factors in HPNE and PDAC cell lines. N, normal.

(*p<0.05, **p<0.01, ****p<0.001)

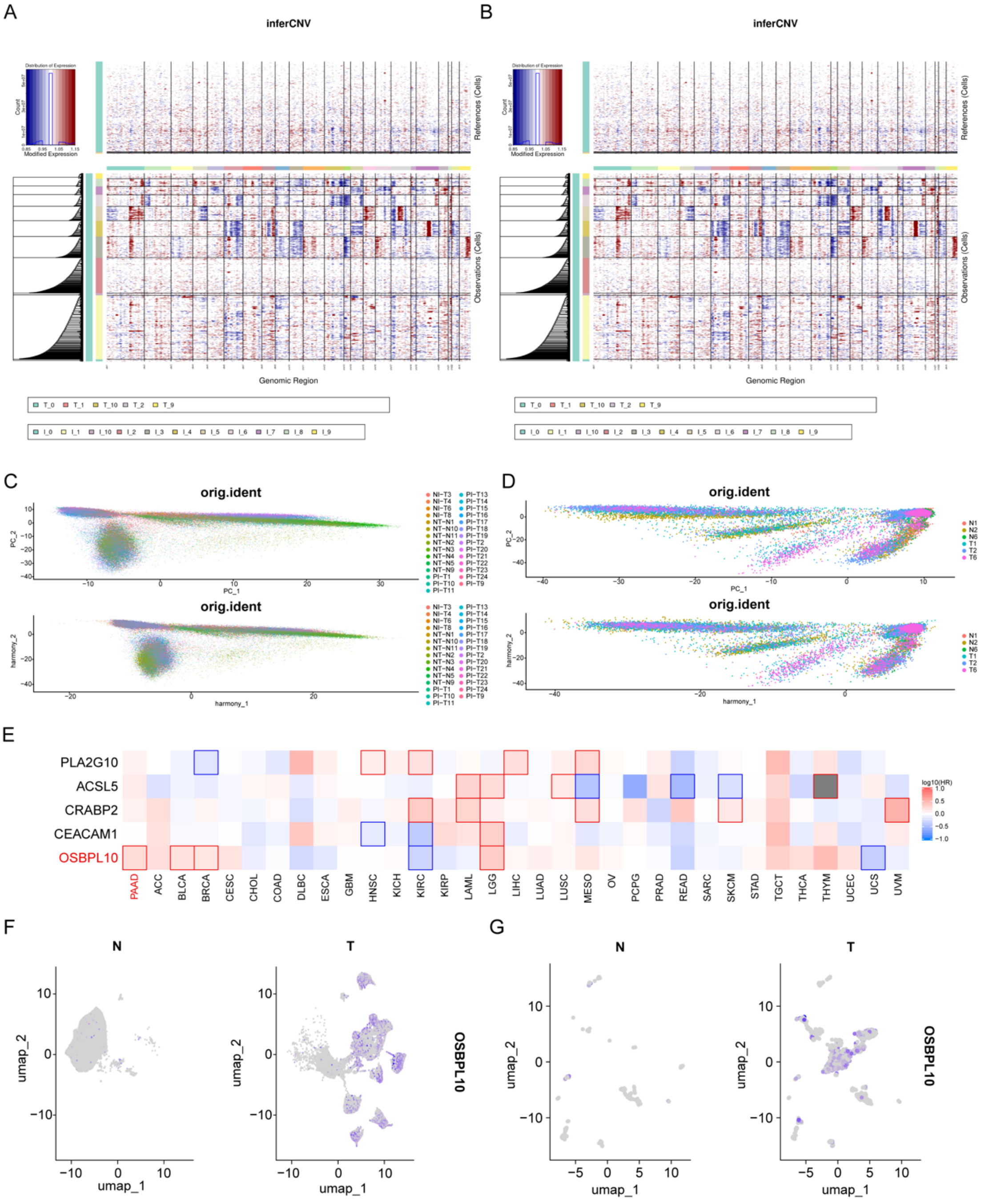

Supplementary Figure. 3 Quality control of PDAC scRNA-seq datasets and expression characteristics of *OSBPL10*.

A-B, Copy number analysis and quality control of datasets CRA001160 (A) and GSE212966 (B).

C-D, Quality control of datasets CRA001160 (C) and GSE212966 (D).

E, Correlation between survival and expression of genes from the intersections of (Fig. 2H) according to TCGA database.

F-G, *OSBPL10* expression in datasets CRA001160(F) and GSE212966(G).

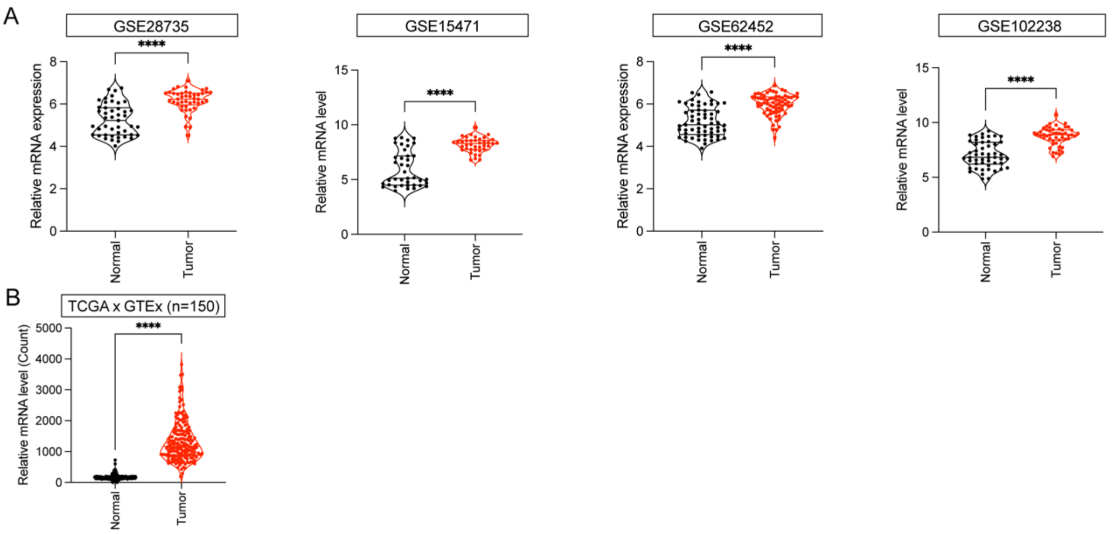

Supplementary Figure. 4 Expression profiles of the *OSBPL10* in GEO and TCGA databases.

A-B, *OSBPL10* expression in GEO databases (A, GSE28735, GSE15471, GSE62452, GSE102238) and TCGA database (B).

(****p<0.001)

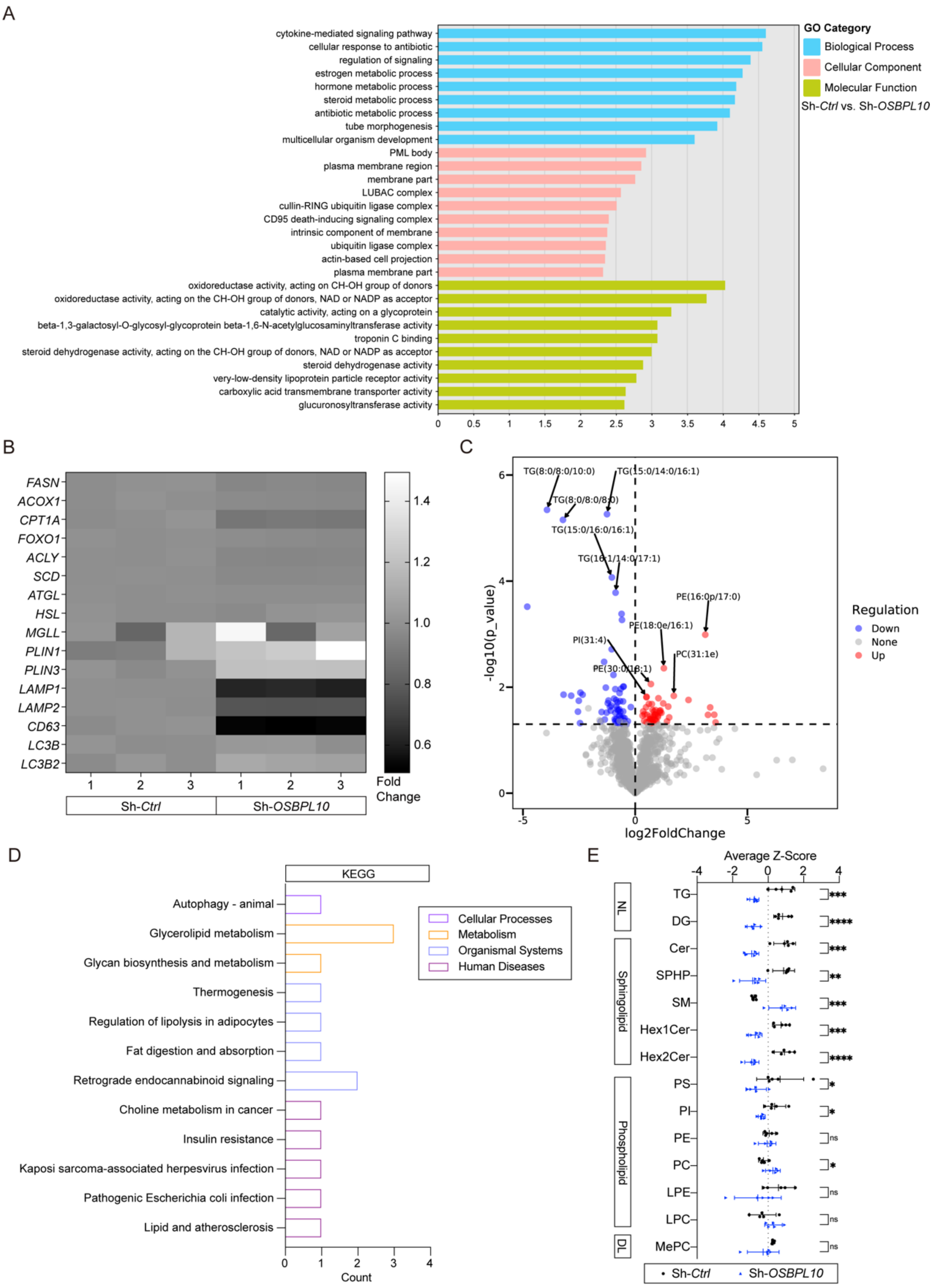

Supplementary Figure. 5 Transcriptomic and lipidomic profiling of *OSBPL10* knockdown in PDAC cells.

A, GO analysis of the mRNA-seq results between the control group and the *OSBPL10*^{KD} group.

B, Expression results in mRNA-seq of indicators related to autophagy and fatty acid metabolism between the control group and the *OSBPL10*^{KD} group.

C, Volcano plot of metabolites in liquid chromatography-mass spectrometry assays results between the control group and *OSBPL10*^{KD} group.

D, KEGG analysis of lipid liquid chromatography-mass spectrometry assays results between the control group and *OSBPL10*^{KD} group.

E, Average Z-score of metabolites in liquid chromatography-mass spectrometry assays results between the control group and *OSBPL10*^{KD} group (n=5, |log2FC|>0.6, $p<0.05$, mean ± SEM.; two-tailed unpaired t test).

(ns, not significant, *$p<0.05$, **$p<0.01$, ***$p<0.005$, ****$p<0.001$)

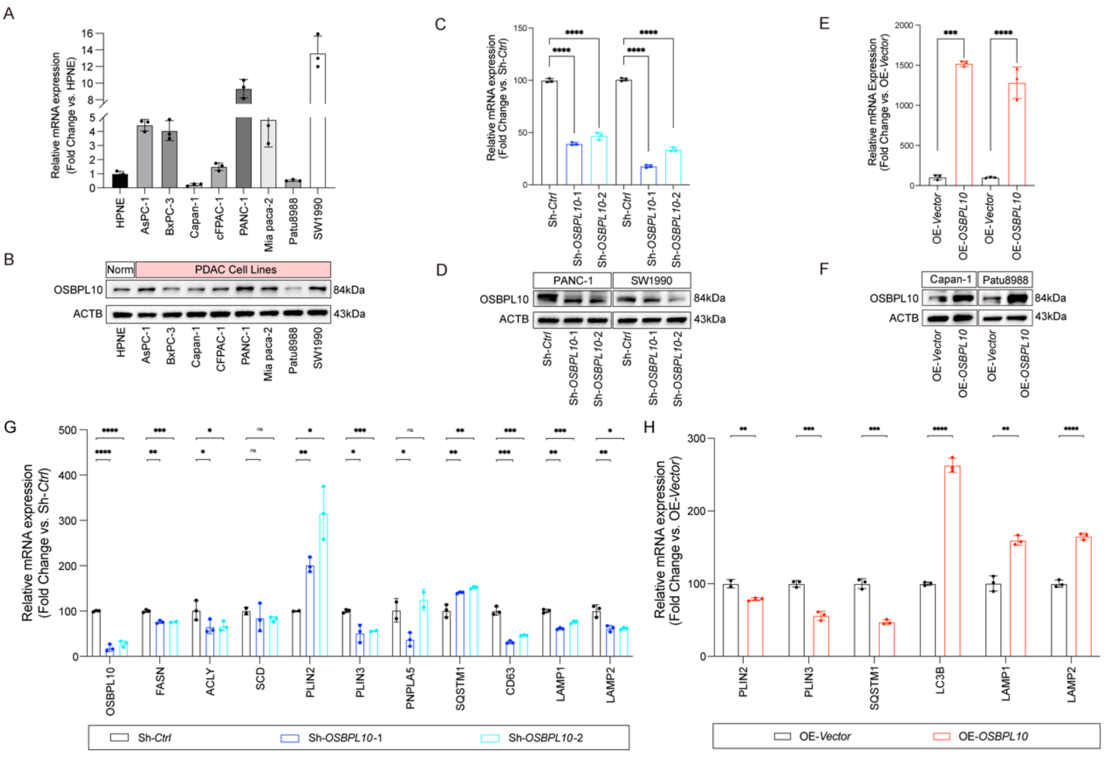

Supplementary Figure. 6 Construction of *OSBPL10* stable cell lines and its effect on the transcription of autophagy- and metabolism-related genes.

A-B, Relative mRNA expression (A) and immunoblotting (A) of HPNE and 8 other

PDAC cell lines. Norm, normal pancreatic ductal cells (n=3, mean ± SEM.; two-tailed unpaired t test).

C-D, Relative mRNA expression (C) and immunoblotting (D) of *OSBPL10* knockdown in PANC-1 and SW1990 (n=3, mean ± SEM.; two-tailed unpaired t test).

E-F, Relative mRNA expression (E) and immunoblotting (F) of *OSBPL10* overexpression in Capan-1 and Patu8988 (n=3, mean ± SEM.; two-tailed unpaired t test).

G-H, Relative mRNA expression in *OSBPL10*^{KD} and *OSBPL10*^{OE} cells about indicators in autophagy and fatty acid metabolism (n=3, mean ± SEM.; two-tailed unpaired t test). (ns, not significant, *p<0.05, **p<0.01, ***p<0.005, ****p<0.001)

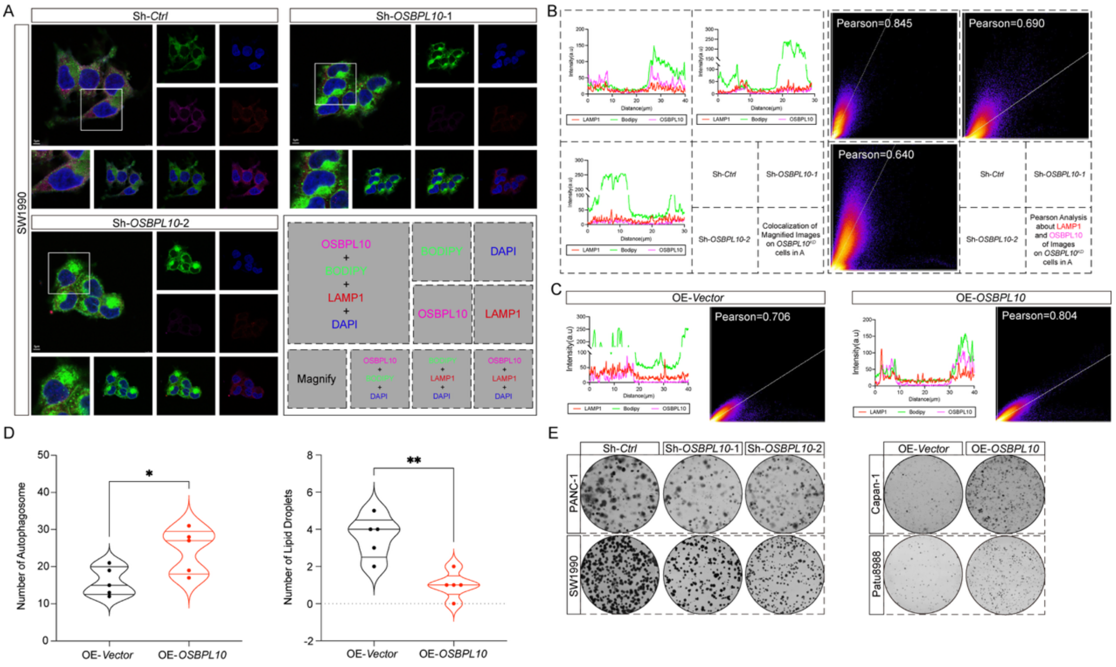

Supplementary Figure. 7 Analysis of *OSBPL10* expression levels, lipophagic activity, and cell proliferation in PDAC cell lines *in vitro*.

A, Representative images of immunofluorescence staining of OSBPL10, LAMP1 and Bodipy in *OSBPL10*^{KD} cell line (n=5, 3 random fields assessed per sample). Blue, DAPI; Green, BODIPY; Red, LAMP1. Pink, OSBPL10. Scale bar, 5μm.

B, Quantification of the colocalization of LAMP1 and BODIPY, and the correlation analysis between LAMP1 and OSBPL10 of *OSBPL10*^{KD} cell line in (A).

C, Quantification of the colocalization of LAMP1 and BODIPY, and the correlation analysis between LAMP1 and OSBPL10 of *OSBPL10*^{OE} cell line in (Fig. 3D).

D, Quantification of autophagosomes and LDs in TEM results between the control group and *OSBPL10*^{OE} group (n=3, 3 random fields assessed per sample, mean ± SEM.; two-tailed unpaired *t* test).

E, Colony formation of *OSBPL10*^{KD} and *OSBPL10*^{OE} cells.

(*p<0.05, **p<0.01, ***p<0.005, ****p<0.001)

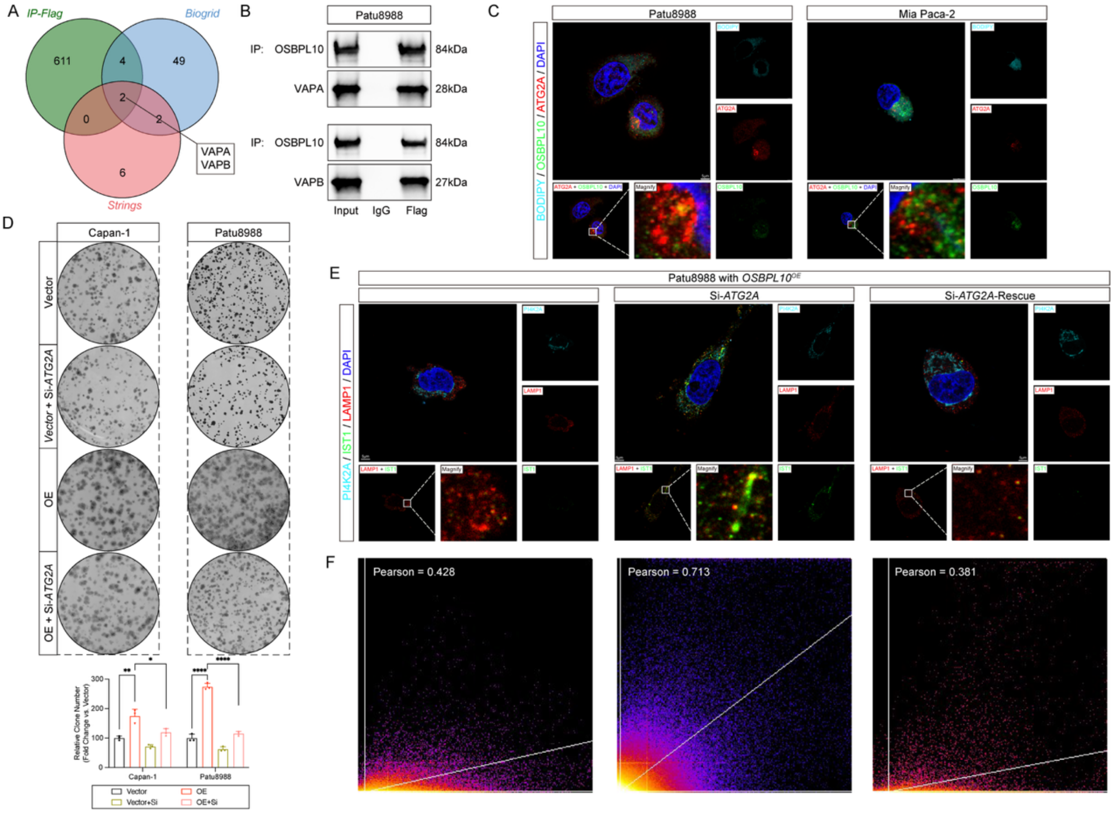

Supplementary Figure. 8 Interaction network analysis of OSBPL10 and verification of ATG2A-mediated lysosomal repair.

A, Venn diagram demonstrating the intersections between the mass spectrometry assay results and the prediction results of protein interacted with OSBPL10 in Biogrid and Strings.

B, Immunoblotting of co-immunoprecipitation of OSBPL10 and VAPA/VAPB in

OSBPL10^{OE} cell line.

C, Representative images of immunofluorescence staining of OSBPL10, ATG2A and BODIPY in Patu8988 and Mia Paca-2 wild type PDAC cell lines (n=5, 3 random fields assessed per sample). Blue, DAPI; Green, OSBPL10; Red, ATG2A. Cyan, BODIPY. Scale bar, 5μm.

D, Colony formation assays of *OSBPL10*^{OE} cells with si-*ATG2A* (n=3, mean ± SEM.; two-tailed unpaired t test).

E, Representative images of immunofluorescence staining of PI4K2A, LAMP1 and IST1 in *OSBPL10*^{OE} cell line with si-*ATG2A* and *ATG2A*-cDNA (n=5, 3 random fields assessed per sample). Blue, DAPI; Green, IST1; Red, LAMP1. Cyan PI4K2A. Scale bar, 5μm.

F, The correlation analysis between IST1 and LAMP1 in (E).

(ns, not significant, *p<0.05, **p<0.01, ***p<0.005, ****p<0.001)

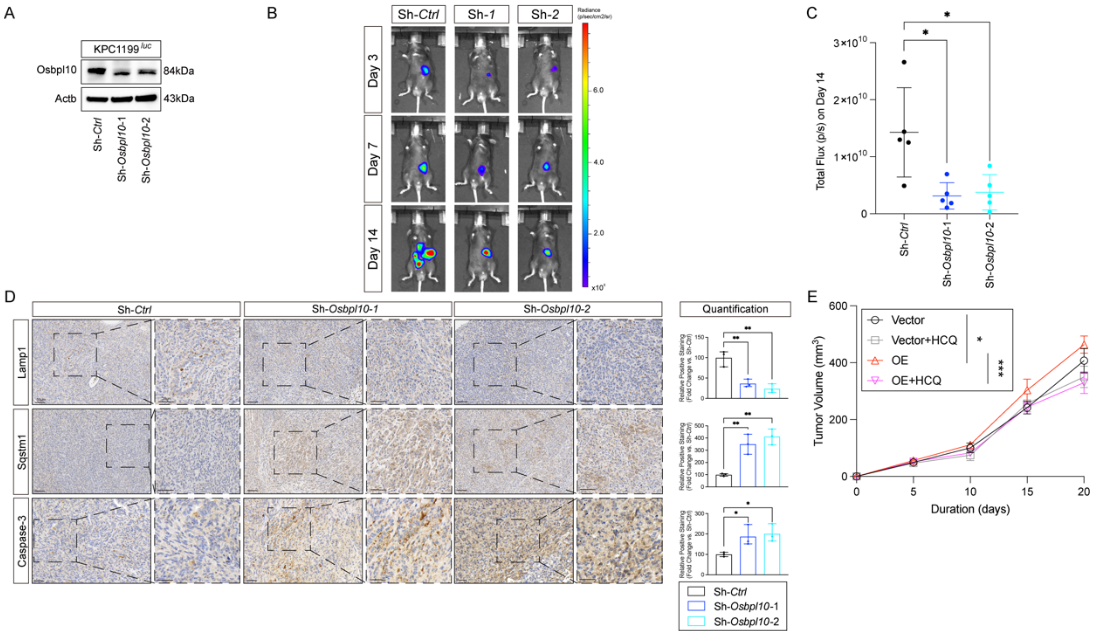

Supplementary Figure. 9 *In vivo* bioluminescence imaging and histopathological analysis of PDAC xenografts.

A, Immunoblotting of the *Osbpl10* knockdown in KPC1199^{luc} cell line.

B, Representative luciferase live images of mice during certain time points of the orthotopic xenografts with *Osbpl10*^{KD} cells (n=5).

C, Quantification of total flux of mice in (B) at Day 14 (n=5, mean ± SEM.; two-tailed unpaired t test).

D, Representative images and quantification of immunohistochemical staining of lamp1, Sqstm1 and Caspase-3 from the orthotopic xenografts with $Osbpl10^{KD}$ cells (n=3, 3 fields assessed per sample, mean ± SEM.; two-tailed unpaired t test). Scale bar, 50μm and 20μm.

E, Tumor volumes of the subcutaneous xenografts with $OSBPL10^{OE}$ cells and the CQ group.

(*$p<0.05$, **$p<0.01$)

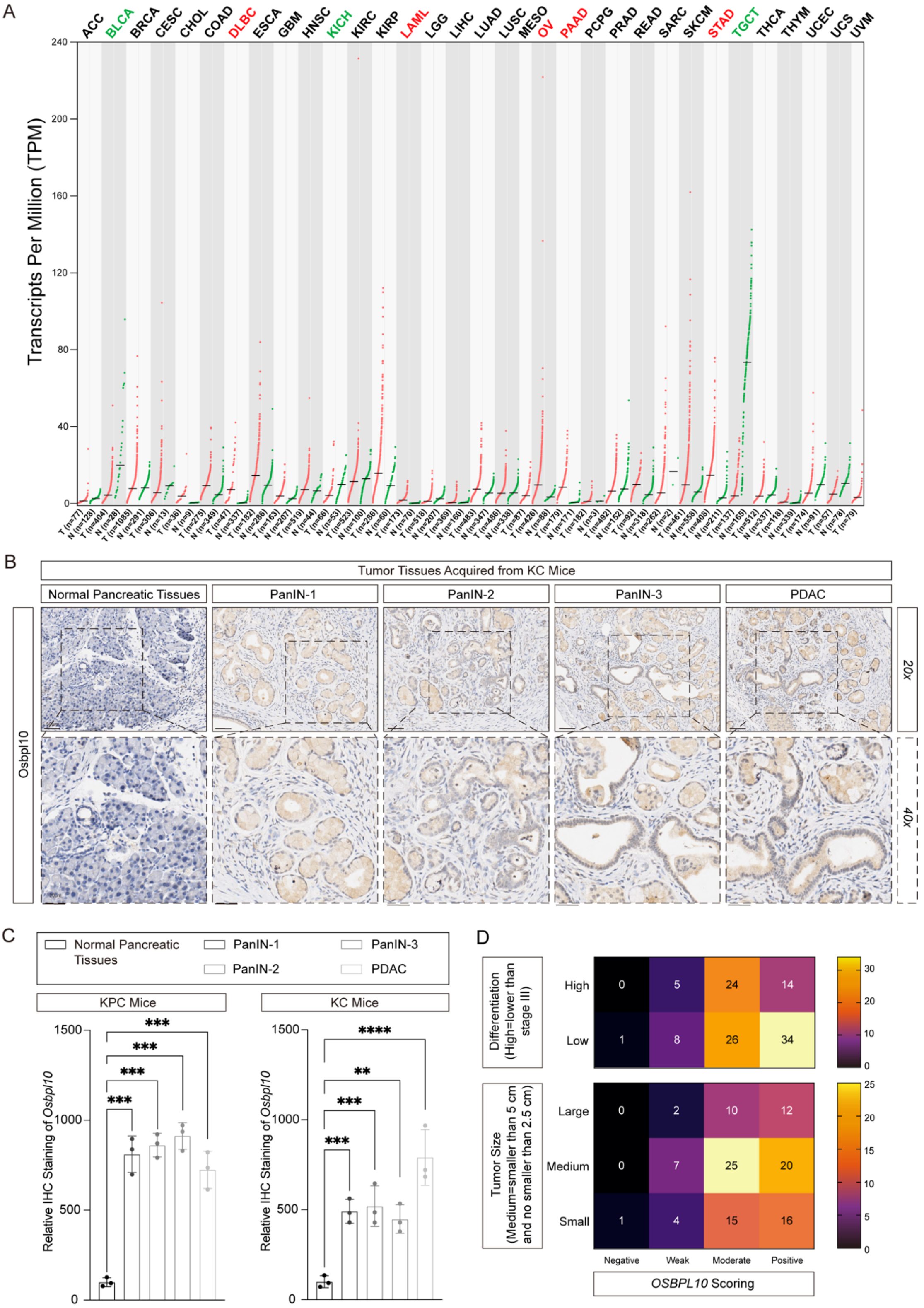

Supplementary Figure. 10 Expression of *OSBPL10* in TCGA solid tumors and quantification of Osbpl10 immunohistochemistry in KPC/KC mice.

A, *OSBPL10* expression in all solid tumors in TCGA database.

B, Representative images of immunohistochemical staining and relative quantification of Osbpl10 in KC mice. Scale bar, 50μm and 20μm.

C, Relative quantification of Osbpl10 in Fig. 7B, n=3, 3 radom fields assessed per sample, mean ± SEM.; two-tailed unpaired t test.

D, Relative quantification of Osbpl10 in KC mice in (B), n=3, 3 radom fields assessed per sample, mean ± SEM.; two-tailed unpaired t test.

(****p*<0.005, *****p*<0.001)

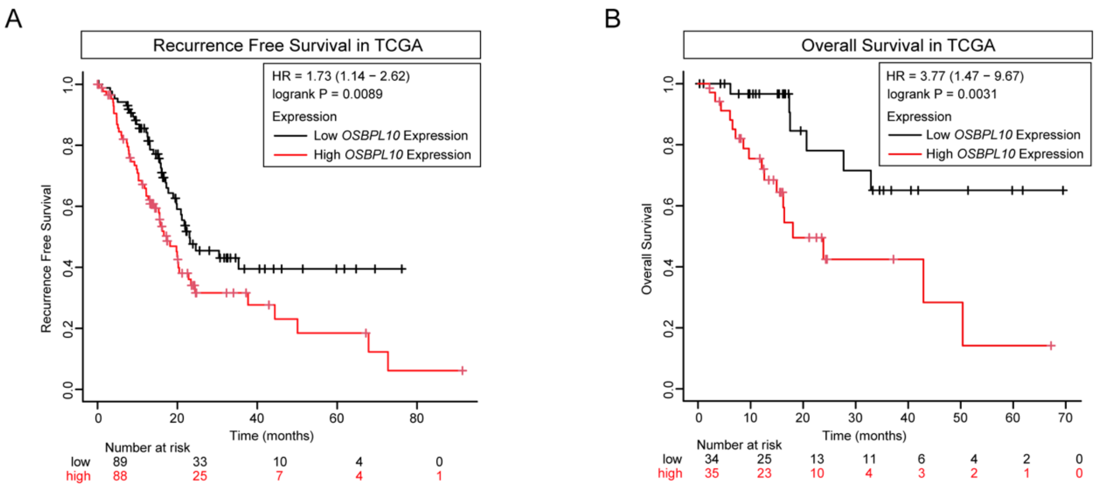

Supplementary Figure. 11 Kaplan-Meier survival analysis of *OSBPL10* expression in the TCGA cohort.

A-B, Kaplan-Meier analysis about the overall survival (A) and recurrence-free survival (B) of patients with high and low *OSBPL10* expression patients.

Supplementary Information 1

The sequence for the overexpression assays of *OSBPL10*

ATGGAGAGGGCAGTCCAGGGCACAGACGGCGGCGGGGGTAGCAACAGCA
GCAGCCGCAGCAGCAGCCGTGCTACCTCGGCGGGCTCCTCGCCCTCCTGCT
CTCTGGCGGGCCGGGGGGTCTCCAGCCGGTCGGCGGCGGCCGGGCTCGGC
GGCGGGGGAAGCCGCAGCAGCCCGGGCTCTGTGGCCGCTAGCCCGTCCGG
GGGAGGCGGCCGCAGGAGGGAGCCGGCGCTCGAGGGCGTGCTCAGCAAA
TACACCAACCTCCTCCAGGGCTGGCAGAACAGGTACTTCGTACTGGATTTC
GAGGCTGGCATCCTGCAGTATTTTGTGAATGAGCAAAGCAAACACCAGAA
GCCTCGAGGAGTCCTGTCTTTATCTGGAGCCATAGTGTCCCTGAGCGATGA
AGCTCCCCACATGCTGGTGGTGTACTCTGCTAATGGAGAGATGTTTAAACT
GAGAGCTGCTGATGCAAAAGAGAAACAATTCTGGGTGACTCAGCTTCGAG
CTTGTGCCAAATACCACATGGAAATGAATTCTAAGAGTGCTCCAAGCTCCC
GAAGCCGAAGTCTCACTTTGCTCCCACATGGAACACCCAATTCTGCGTCTC
CCTGTAGCCAGAGACACCTCAGTGTGGGGGCCCCCGGTGTTGTCACAATC
ACGCATCACAAGTCGCCTGCAGCCGCCCGAAGAGCCAAGAGTCAGTATTC
CGGCCAGCTTCACGAAGTCAGAGAGATGATGAACCAGGTGGAAGGGCAG
CAGAAGAACCTTGTGCACGCCATTGAGTCCCTGCCAGGGTCCGGCCCCCT
CACTGCCTTGGACCAGGACCTGCTGCTCCTGAAAGCTACCTCTGCTGCCAC
CCTCAGCTGCCTTGGGGAGTGCCTCAACTTGTTACAGCAGAGTGTGCACCA
GGCGGGCCAGCCCAGCCAGAAGCCAGGAGCCTCGGAAAACATCCTGGGAT
GGCACGGGTCCAAGTCACATTCCACAGAGCAGCTGAAAAATGGGACACTT
GGCTCTTTGCCATCAGCCAGTGCCAACATAACCTGGGCAATTTTACCAAAC
TCTGCTGAAGACGAACAAACCTCACAGCCAGAGCCAGAGCCAAACTCAG
GCTCTGAATTGGTTTTGTCTGAAGATGAAAAAAGTGACAATGAAGATAAGG
AAGAGACGGAATTGGGCGTCATGGAGGATCAGCGTAGTATAATTCTTCATC
TCATTTCACAACTCAAACTTGGAATGGATTTGACCAAGGTGGTGCTTCCCA
CCTTTATCCTGGAGAAGCGATCTTTGCTGGAGATGTATGCAGATTTCATGGC
GCACCCAGACCTACTGCTGGCCATCACCGCTGGGGCCACACCAGAGGAGA
GAGTCATTTGCTTCGTTGAGTATTATCTCACAGCCTTTCACGAGGGCCGCAA

GGGCGCTTTAGCCAAGAAGCCCTACAACCCCATCATAGGCGAGACATTTCA
CTGCTCCTGGGAAGTTCCCAAGGACAGGGTCAAGCCTAAGAGGACTGCTT
CCCGCTCTCCTGCCAGCTGTCACGAACACCCAATGGCCGATGACCCTTCCA
AAAGCTACAAACTAAGGTTTGTGGCTGAGCAAGTGTCCCATCACCCACCC
ATCTCCTGCTTCTACTGTGAGTGCGAGGAGAAGAGACTGTGTGTCAACACT
CATGTATGGACCAAAAGCAAGTTCATGGGCATGTCCGTGGGGGTCTCTATG
ATAGGGGAAGGTGTGTTGAGGCTCCTGGAACACGGGGAGGAGTACGTATT
CACCCTGCCTAGTGCCTACGCCCGGTCCATTCTCACCATCCCATGGGTGGA
GCTCGGAGGAAAAGTCAGCATCAACTGTGCCAAGACTGGGTACTCAGCGA
CAGTGATATTCCACACGAAGCCTTTCTATGGAGGGAAAGTCCACAGGGTTA
CCGCAGAAGTGAAGCACAACCCAACCAACACCATTGTTTGTAAAGCCCAT
GGGGAATGGAATGGTACTTTAGAGTTCACCTACAACAATGGAGAAACCAA
AGTCATCGACACAACCACACTGCCAGTGTATCCCAAGAAGATCAGACCTCT
TGAGAAGCAGGGACCCATGGAGTCCAGGAACCTCTGGCGGGAGGTGACC
CGATACCTGCGGCTGGGGGACATTGACGCAGCCACCGAGCAGAAGCGGCA
CCTAGAGGAGAAGCAACGGGTGGAGGAACGGAAGCGCGAGAACCTCCGC
ACACCATGGAAGCCCAAATATTTATCCAGGAGGGCGATGGCTGGGTATAC
TTCAATCCCCTCTGGAAAGCACACTAG

Supplementary Information 2

The sequences of the knockdown assays of *Osbpl10*

Sh-*Osbpl10*-1 CCGAAGAGCCAAGAGTCAGTA

Sh-*Osbpl10*-2 TCCGCCAGTGCCAACATAACA

Sh-*Osbpl10*-3 CGCCAGAGGAGAGAGTCATTA

The sequences of the knockdown assays of *OSBPL10*

Sh-*OSBPL10*-1 GACAGTGATATTCCACACGAA

Sh-*OSBPL10*-2 GTCATTTGCTTCGTTGAGTAT

Sh-*OSBPL10*-3 CCAACATAACCTGGGCAATTT

The sequences of the cDNA of *ATG2A*

ATGTCACGATGGCTGTGGCCATGGTCAAACTGTGTGAAAGAGCGGGTCTG
CCGCTACTTGCTGCACCACTACTTAGGTCACTTCTTCCAAGAGCACCTCAG
CCTGGACCAGCTCAGCCTCGATCTGTACAAGGGCAGCGTTGCCCTGCGAG
ACATCCACCTGGAAATCTGGTCTGTGAACGAGGTGCTGGAGTCAATGGAG
TCACCGCTGGAGCTGGTGGAAGGCTTCGTGGGCTCCATCGAGGTGGCCGT
GCCCTGGGCTGCTCTGCTCACCGACCACTGCACAGTGCGCGTGTCCGGCCT
CCAGCTCACCTTGCAGCCCCGCCGGGGTCCAGCGCCAGGGGCTGCCGACT
CACAGAGCTGGGCCTCATGCATGACCACAAGCCTGCAGCTGGCCCAGGAG
TGTCTGCGGGATGGGCTACCGGAGCCCTCTGAGCCACCACAGCCCCTGGA
GGGGCTGGAGATGTTTGCCCAGACCATTGAGACTGTGCTTCGGAGGATCA
AAGTGACCTTCCTGGACACTGTCGTGAGGGTGGAGCACTCTCCGGGTGAT
GGGGAACGTGGTGTGGCCGTCGAGGTCCGTGTGCAGAGACTGGAGTACTG
TGATGAGGCAGTGCGGGACCCAAGCCAGGCGCCGCCGGTGGACGTGCATC
AGCCGCCTGCCTTCCTGCACAAGCTGCTGCAGCTGGCAGGGGTCCGCCTG
CACTACGAGGAGCTCCCGGCACAGGAAGAGCCTCCAGAGCCCCCCTTGCA
GATCGGCAGCTGCTCAGGGTACATGGAGCTGATGGTGAAGTTGAAGCAAA
ATGAGGCCTTCCCTGGCCCCAAGTTGGAGGTGGCGGGACAGCTGGGCTCC

CTGCACCTGCTCCTGACCCCGAGGCAGCTCCAGCAACTTCAGGAACTGCT
CAGCGCCGTGAGCCTTACAGACCACGAGGGCCTGGCTGACAAGCTGAACA
AGAGCCGCCCGCTAGGTGCCGAAGACCTGTGGCTGATTGAGCAGGACCTG
AACCAGCAGCTGCAGGCAGGGGCAGTGGCTGAGCCCCTCAGCCCAGACC
CCCTTACCAACCCCCTTCTCAACCTGGATAACACTGACCTCTTCTTCTCCAT
GGCTGGCCTCACAAGCAGTGTGGCCTCAGCCCTCTCTGAGCTCTCCCTCTC
CGATGTAGACCTGGCCTCCTCTGTGCGCAGCGACATGGCCTCCCGCCGGCT
CTCTGCCCAGGCCCACCCAGCTGGCAAGATGGCCCCCAACCCCCTCCTGG
ACACCATGCGCCCTGACTCGCTGCTGAAGATGACCTTGGGGGGTGTGACC
CTGACCTTGCTTCAGACGTCTGCCCCATCTTCCGGACCACCTGACCTCGCC
ACGCACTTTTTCACCGAGTTTGATGCCACCAAGGATGGGCCCTTCGGTTCC
CGAGACTTCCATCACCTTCGACCACGCTTCCAGAGGGCCTGTCCCTGTAGC
CATGTTCGGCTAACGGGCACAGCCGTGCAGCTGTCCTGGGAGCTGCGGAC
GGGCAGTCGGGGCCGGCGGACAACCAGCATGGAAGTGCACTTCGGGCAG
CTGGAGGTGCTGGAGTGTCTGTGGCCCCGGGGCACCTCTGAGCCTGAGTA
CACGGAGATCCTGACCTTTCCTGGTACGCTGGGCTCCCAGGCCTCAGCTCG
GCCCTGCGCCCATCTGCGCCACACACAGATCCTGCGCCGTGTGCCTAAGAG
CCGACCCCGGCGCTCAGTTGCCTGCCATTGCCACTCAGAACTGGCCCTGGA
CCTGGCCAACTTCCAGGCGGACGTGGAGCTGGGGGCCCTGGACCGGCTGG
CCGCCCTACTGCGCCTGGCCACCGTACCTGCTGAGCCTCCAGCCGGCCTGC
TGACAGAGCCCCTGCCGGCGATGGAGCAGCAGACGGTATTTCGGCTCTCTG
CACCCCGGGCCACGCTGCGGCTGCGCTTCCCCATTGCCGACCTGCGGCCTG
AGCCGGACCCCTGGGCGGGCCAGGCCGTGCGGGCTGAGCAGCTTCGGCTG
GAGCTGAGTGAGCCCCAGTTCCGGTCAGAGCTTAGCAGTGGGCCTGGTCC
CCCAGTCCCCACCCACCTGGAACTCACCTGCTCCGACCTACATGGTATCTAT
GAAGATGGAGGGAAGCCACCTGTCCCTTGCCTGCGTGTCTCCAAAGCCCT
GGACCCCAAGAGCACTGGGCGCAAGTACTTCCTGCCCCAGGTAGTGGTGA
CTGTGAACCCCCAGTCCAGCAGCACACAGTGGGAGGTGGCCCCGGAGAA
GGGAGAGGAACTGGAGCTGTCAGTGGAGAGTCCCTGTGAGCTGCGGGAA
CCTGAGCCCTCGCCCTTCTCCTCTAAGAGGACCATGTATGAGACAGAGGAG

ATGGTGATCCCTGGAGACCCTGAGGAGATGAGGACGTTCCAGAGCCGGAC
CCTGGCACTGTCCCGCTGCAGCCTGGAAGTGATCCTGCCCAGTGTCCACAT
CTTTCTGCCCAGCAAGGAGGTCTACGAGAGCATCTACAACAGGATCAACA
ACGACCTGCTCATGTGGGAGCCTGCAGATCTGCTTCCCACCCCCGACCCCG
CCGCCCAGCCCTCGGGCTTCCCCGGCCCCTCAGGCTTCTGGCACGACAGCT
TTAAGATGTGCAAGTCAGCCTTCAAGCTGGCCAACTGCTTTGATCTCACCC
CAGACTCGGACTCGGATGACGAGGATGCCCACTTCTTCTCAGTGGGGGCAT
CAGGTGGCCCACAGGCCGCTGCCCCTGAGGCCCCAAGTCTTCACTTGCAG
AGCACCTTCTCTACACTGGTGACAGTGCTGAAGGGGCGGATCACAGCCCT
CTGTGAGACCAAGGATGAGGGTGGGAAGCGGCTGGAGGCTGTGCACGGG
GAGCTGGTGCTGGACATGGAGCACGGTACCCTCTTCAGCGTCTCCCAGTAC
TGTGGCCAGCCAGGACTTGGCTACTTCTGTCTGGAAGCTGAAAAGGCAAC
ACTCTACCACCGAGCGGCCGTGGATGACTACCCGCTGCCCAGTCACCTGGA
CCTTCCCAGTTTCGCTCCCCCGGCTCAGCTGGCCCCAACCATCTACCCATCG
GAGGAAGGGGTGACCGAGCGGGGAGCCTCGGGCCGCAAGGGCCAGGGCC
GGGGACCCCACATGTTGTCCACTGCTGTGCGCATCCACCTGGACCCCCACA
AGAATGTGAAGGAGTTCCTGGTGACTCTCCGCTTACATAAGGCCACCTTGC
GCCACTACATGGCCCTGCCCGAGCAGAGCTGGCATTCCCAGTTGTTGGAGT
TCCTAGACGTGCTGGATGACCCTGTGCTGGGCTACCTGCCCCCGACGGTCA
TCACCATCCTGCACACACACCTGTTCTCCTGCTCTGTGGACTATAGGCCACT
CTACCTCCCAGTGCGTGTCCTCATCACCGCGGAGACCTTCACTCTCTCCAG
CAACATCATCATGGACACCTCCACCTTCCTGCTCAGGTTCATCCTCGATGAC
TCCGCCTTGTACCTGTCCGACAAGTGTGAGGTGGAGACCCTGGACCTGCG
GCGAGATTATGTCTGTGTTTTGGATGTTGACCTCTTGGAACTTGTGATTAAA
ACCTGGAAAGGGAGCACCGAGGGCAAACTGAGCCAGCCACTATTCGAGCT
GCGCTGCTCCAACAATGTGGTACACGTGCACAGCTGTGCCGACTCCTGTGC
CCTGCTGGTCAACCTGCTCCAGTACGTAATGAGCACAGGCGATCTGCACCC
CCCACCCCGGCCCCCCAGCCCCACGGAGATCGCCGGCCAGAAGCTCTCGG
AGAGTCCTGCCTCTCTGCCCTCGTGCCCCCCAGTGGAGACGGCCCTCATCA
ACCAGCGTGACCTGGCCGACGCCCTCCTGGACACCGAGCGCAGCCTACGG

GAGCTGGCCCAGCCTTCAGGTGGCCACCTCCCTCAGGCGTCGCCCATCTCC
GTCTACCTATTCCCAGGTGAACGGAGTGGGGCCCCACCCCCTTCACCACCT
GTCGGGGGCCCTGCTGGCAGCTTAGGGTCATGCTCAGAGGAGAAGGAAGA
TGAAAGGGAAGAGGAGGGCGATGGAGACACCCTGGACAGTGATGAGTTC
TGCATCCTTGATGCTCCCGGCCTGGGCATCCCGCCCCGAGATGGGGAGCCT
GTGGTGACACAGCTGCATCCCGGCCCCATCGTTGTGAGGGACGGTTACTTC
TCACGGCCGATCGGCAGCACGGACTTGCTGCGGGCACCTGCCCATTTCCCA
GTGCCCAGCACTCGGGTGGTGCTACGTGAGGTCTCCCTCGTCTGGCACCTC
TATGGGGGCCGAGACTTTGGCCCCCACCCCGGCCACAGGGCAAGAACTGG
CCTCTCAGGTCCCAGGAGCTCCCCTTCCCGCTGCTCTGGCCCCAACCGGCC
CCAGAACTCATGGCGCACGCAGGGGGGCAGCGGGCGGCAGCACCATGTCC
TCATGGAGATCCAGCTGAGCAAGGTAAGCTTCCAGCACGAGGTGTACCCA
GCGGAGCCAGCCACAGGCCCTGCGGCCCCCAGCCAGGAGCTGGAGGAGC
GACCGCTGTCCCGTCAGGTGTTCATCGTGCAGGAGCTGGAGGTCCGAGAC
CGGCTCGCCTCCTCCCAGATCAACAAGTTCCTGTACCTACACACGAGTGAG
CGGATGCCGCGACGTGCCCACTCTAACATGCTCACCATCAAAGCGCTGCAT
GTGGCCCCCACTACCAACCTGGGTGGGCCTGAGTGCTGTCTCCGCGTCTCG
CTGATGCCCCTGCGGCTCAATGTGGACCAGGATGCCCTCTTCTTCCTCAAG
GACTTCTTCACTAGTCTGGTGGCCGGCATCAACCCCGTGGTCCCAGGGGAG
ACCTCCGCTGAGGCTCGCCCCGAGACTCGAGCCCAGCCCAGCAGCCCCCT
GGAAGGGCAGGCCGAAGGCGTAGAGACCACTGGTTCGCAGGAGGCCCCA
GGAGGTGGACACAGCCCCTCCCCTCCTGACCAGCAGCCCATCTACTTCAGA
GAGTTCCGCTTCACGTCTGAGGTCCCCATCTGGCTGGATTACCATGGCAAG
CACGTCACGATGGACCAGGTGGGCACTTTTGCTGGCCTCCTCATCGGCCTG
GCCCAACTCAACTGCTCCGAGCTGAAGCTAAAGCGGCTCTGTTGCAGGCA
CGGGCTCCTGGGTGTGGACAAGGTGCTGGGCTATGCCCTCAACGAGTGGC
TGCAGGACATCCGCAAGAACCAGCTGCCCGGCCTGCTGGGAGGCGTGGGC
CCCATGCACTCGGTTGTCCAGCTCTTCCAAGGGTTCCGGGACCTGCTGTGG
CTGCCCATTGAGCAGTACAGGAAGGATGGCCGCCTCATGCGGGGGCTGCA
GCGAGGGGCTGCCTCCTTTGGCTCATCCACAGCCTCTGCCGCCCTGGAACT

CAGCAACCGGTTGGTACAGGCTATCCAGGCCACAGCTGAGACCGTGTATGA

CATCCTGTCCCCGGCAGCCCCCGTCTCCCGCTCCCTGCAGGATAAGCGCTC

TGCGCGGAGGCTGCGCAGGGGCCAGCAGCCTGCCGACCTGCGGGAGGGT

GTGGCCAAGGCCTACGACACAGTGCGAGAGGGCATCTTGGATACAGCTCA

GACCATCTGTGACGTGGCATCGCGGGGCCATGAGCAGAAGGGGCTGACGG

GCGCCGTGGGGGGCGTGATCCGCCAGCTGCCCCCGACTGTGGTGAAGCCG

CTCATCCTGGCCACGGAGGCCACGTCCAGCCTGCTCGGGGGCATGCGCAA

CCAGATTGTCCCCGACGCCCACAAGGACCACGCCCTCAAGTGGCGCTCGG

ACAGTGCCCAAGACTGA

Supplementary Table 1 The sequences of target genes primers

Genes	Primers
OSBPL10	F: 5'- GGGGAGTGCCTCAACTTGTTA - 3'
	R: 5'- AGGTTTGTTCGTCTTCAGCAG - 3'
PLIN2	F: 5'- ATGGCATCCGTTGCAGTTGAT- 3'
	R: 5'- GGACATGAGGTCATACGTGGAG - 3'
FASN	F: 5'- AAGGACCTGTCTAGGTTTGATGC - 3'
	R: 5'- TGGCTTCATAGGTGACTTCCA - 3'
CPT1A	F: 5'- CTCCGCCTGAGCCATGAAG - 3'
	R: 5'- CACCAGTGATGATGCCATTCT - 3'
SQSTM1	F: 5'- GACTACGACTTGTGTAGCGTC - 3'
	R: 5'- AGTGTCCGTGTTTCACCTTCC- 3'
CD63	F: 5'- ATGCAGGCAGATTTTAAGTGCT- 3'
	R: 5'- GTTCTTCGACATGGAAGGGATTT- 3'
LAMP1	F: 5'- CAGATGTGTTAGTGGCACCCA - 3'
	R: 5'- TTGGAAAGGTACGCCTGGATG - 3'
LAMP2	F: 5'- TGGCAATGATACTTGTCTGCTG - 3'
	R: 5'- ACGGAGCCATTAACCAAATACAT - 3'
PNPLA5	F: 5'- GGACGCTGGTGTCTAAGGAAC- 3'
	R: 5'- CAGCATCCCAGTTTCCGTCAG- 3'
PLIN3	F: 5'- TCACCATGTTCCGGGACATTG- 3'
	R: 5'- CACCTGGTCCTTCACATTGG- 3'
Human 18s	F: 5'- GGCCCTGTAATTGGAATGAGTC- 3'
	R: 5'- CCAAGATCCAACTACGAGCTT- 3'

Supplementary Table 2 The antibodies used for immunoblotting

Antibodies	Hosts	Manufacturers (Lot Number)	Concentrations
OSBPL10 Polyclonal Antibody	Rabbit	Proteintech (15491-1-AP)	1:800
β-Actin Polyclonal Antibody	Rabbit	Abways (AB0035)	1:10000
PLIN2 Polyclonal Antibody	Rabbit	Proteintech (15294-1-AP)	1:1000
LAMP1 Polyclonal Antibody	Rabbit	Cell Signaling Technology (9091)	1:1000
SQSTM1 Polyclonal Antibody	Rabbit	Cell Signaling Technology (5114)	1:1000
ATG2A Polyclonal Antibody	Rabbit	Proteintech (23226-1-AP)	1:2000
Goat Anti-Rabbit IgG (H+L)	Rabbit	ShareBio (SB-AB0101)	1:10000

Supplementary Table 3 Baseline Clinical Statistics of the Ren Ji cohort

		Overall
Total		130
Age (Years) [mean (SD)]		66.72 (9.66)
Sex	Male	46
	Female	84
Overall Survival (Days) [mean (SD)]		919.08 (677.30)
Survival [mean (SD)]		0.76 (0.43)
Liver Metastasis [mean (SD)]		0.08 (0.48)
Lymph Node Metastasis [mean (SD)]		0.34 (0.48)
Number of Metastatic Lymph Node [mean (SD)]		0.49 (0.98)
Peritoneum Implanting Metastasis [mean (SD)]		0.03 (0.17)
Distant Metastasis [mean (SD)]		0.11 (0.31)
TNM Stage (0=I, II/1=III, IV) [mean (SD)]		2.38 (0.81)
Tumor Differentiation (0=I, II/III, IV) [mean (SD)]		0.43 (0.50)
Tumor Size (0=smaller than 3cm) [mean (SD)]		0.67 (0.47)

Supplementary Table 4 The H-score of *OSBPL10* Expression in the Ren Ji TMA

Patient ID	*OSBPL10* Expression Scores
1	*3*
2	*3*
3	*4*
4	*4*
5	*3*
6	*2*
7	*6*
8	*4*
9	*4*
10	*6*
11	*4*
12	*6*
13	*6*
14	*3*
15	*6*
16	*2*
17	*4*
18	*6*
19	*6*
20	*3*
21	*4*
22	*3*
23	*2*
24	*6*
25	*1*
26	*4*
27	*3*

28	*4*
29	*2*
30	*3*
31	*9*
32	*2*
33	*1*
34	*9*
35	*9*
36	*1*
37	*2*
38	*6*
39	*9*
40	*6*
41	*9*
42	*9*
43	*3*
44	*9*
45	*2*
46	*9*
47	*6*
48	*9*
49	*6*
50	*6*
51	*9*
52	*1*
53	*9*
54	*3*
55	*3*
56	*6*

57	*4*
58	*9*
59	*6*
60	*6*
61	*6*
62	*6*
63	*9*
64	*6*
65	*6*
66	*9*
67	*4*
68	*6*
69	*6*
70	*9*
71	*6*
72	*9*
73	*6*
74	*9*
75	*9*
76	*6*
77	*6*
78	*9*
79	*3*
80	*2*
81	*6*
82	*6*
83	*9*
84	*6*
85	*9*

86	*6*
87	*9*
88	*2*
89	*3*
90	*4*
91	*9*
92	*4*
93	*9*
94	*9*
95	*9*
96	*4*
97	*6*
98	*9*
99	*6*
100	*9*
101	*6*
102	*9*
103	*6*
104	*4*
105	*6*
106	*6*
107	*9*
108	*2*
109	*6*
110	*9*
111	*4*
112	*3*
113	*6*
114	*4*

115	*6*
116	*3*
117	*4*
118	*6*
119	*9*
120	*6*
121	*6*
122	*2*
123	*4*
124	*9*
125	*6*
126	*3*
127	*2*
128	*9*
129	*6*
130	*6*
